# Identification of high blanchability donors, candidate genes and markers in groundnut

**DOI:** 10.1186/s12870-025-07309-9

**Published:** 2025-10-21

**Authors:** Priya Shah, Sunil S. Gangurde, Ramachandran Senthil, Prashant Singam, Ovais Hamid Peerzada, Pasupuleti Janila, Kuldeep Singh, Sean Mayes, Manish K. Pandey

**Affiliations:** 1 Center for Pre-Breeding Research (CPBR), Center of Excellence in Genomics & Systems Biology (CEGSB), International Crops Research Institute for the Semi-Arid Tropics (ICRISAT), Hyderabad, 502324 Telangana India; 2 Department of Genetics, Osmania University, Hyderabad, 500007 Telangana India; 3 CIMMYT-China International Maize and Wheat Research Center, Shandong Agricultural University, Taian, 271000 Shandong China

**Keywords:** Blanchability, Groundnut, Genome-wide association study, markers, Testa

## Abstract

Blanchability is the ability of seeds to shed their seed coat (testa) and is a trait of economic importance in the food processing industry, yet remains underexplored in breeding programs. In this study, blanchability was evaluated in 184 groundnut accessions from the ICRISAT minicore collection to identify associated genomic regions, candidate genes, and molecular markers. Significant variability was observed over two seasons, with values ranging from 3.98 to 70.08%. Ten genotypes, including ICG10890, ICG9507, ICG13982, and ICG297, showed high blanchability, with ICG297 emerging as a promising donor based on cluster analysis of blanchability and agronomic traits. Genome-wide associations study (GWAS) using the 58 K ‘Axiom_*Arachis*’ SNP array revealed 58 significant SNP-trait associations, highlighting important genes such as *isocitrate dehydrogenase* and *ubiquitin ligase*, which influence seed coat structure and cell wall integrity thereby affecting blanchability. Further, nine SNPs were selected via allele mining, among these four SNPs, on chromosomes A01 (snpAH00551, *AhBL01*), A06 (snpAH00554, *AhBL02*), B04 (snpAH00558, *AhBL03*), and B07 (snpAH00559, *AhBL04*), effectively distinguishing between high and low blanchability genotypes. These validated SNPs present valuable tools for genomics-assisted breeding. Overall, the finding contributes towards better understanding of the genetic basis of blanchability in groundnut, providing key genomic resources for improving processing-related traits.

**Supplementary Information:**

The online version contains supplementary material available at 10.1186/s12870-025-07309-9.

## Introduction

Groundnut (*Arachis hypogaea* L.) also known as peanut, is a major leguminous crop, grown mostly as a food and feed crop globally [1]. The cultivated species of groundnut is a self-pollinated allotetraploid crop (2n = 4x = 40) with 2.54 Gb genome size [2–4] and belongs to the *Fabaceae* family [5, 6]. It is a key seasonal Herbaceous legume crop grown in arid and semi-arid regions and is recognized as one of the important sources of edible oil and protein. Groundnut kernels contain 16–36% of protein, 36–54% oil and between 10% and 20% of total carbohydrates and has secured 13^th^ position among the most vital food crops and ranked 4^th^ in the list of the most vital oilseed crops [7, 8]. It has significant market value and enormous nutritional value of high mono-unsaturated fatty acid (MUFA) in oil content and possess significant amounts of vitamins and minerals [9]. Additionally, it also possesses several nutritional qualities such as soluble sugars and possesses rich amounts of calcium (Ca), iron (Fe), Vitamin B and E (tocopherol). Furthermore, anti-nutrients like trypsin inhibitor and phytic acid are present in groundnut that may be deactivated by the process of boiling and roasting [10, 11].

According to industrial importance, edible food products derived from groundnut include raw or roasted nuts, refined oil, groundnut butter, salted kernels, groundnut flour, and various confectionery items. During the processing of groundnut to prepare the edible products, an important operation is the separation of the testa or seed coat (skin) and the process is called blanching. The blanchability is the capacity of a genotype to release its testa very easily. This characteristic is of great economic value in the processing of groundnut raw and processed food products since it reduces the cost of food processing. Additionally, this trait also holds importance in terms of quality control as blanchability leads to removal of damaged kernels, including aflatoxin contamination. Moreover, in terms of processing applications, globally, about 48% of groundnuts are used for the preparation of raw and processed food products such as groundnut milk, groundnut butter, groundnut chikki, groundnut cake, salted and roasted groundnut, groundnut bar chocolate, chutney, while oil extraction accounts for 52% usage. However in India, the groundnut utility accounts for food, seed, and oil extraction is 44%, 24% and 30% respectively [12, 13].

Blanchability has enormous economic significance in the production of food products made from groundnut. If the groundnut variety has low blanchability, the processing of the product becomes laborious, as it results in high re-processing and thus higher costs and time to get marketable products. This trait is associated with the seed coat and its structural properties as well as its chemical composition, such as lignin, pectin, and polyphenols. Blanchability is reported to be a trait with high heritability and genetically regulated, hence the breeding could help in improving the groundnut cultivars with optimum blanchability (~ 75%) [14]. In this context, GAB (Genomics-assisted breeding) holds higher ability for accelerated improvement of crop varieties as the conventional plant breeding is prolonged and laborious. Though there are several studies reported on germplasm screening for blanchability [14, 15], despite that only one study has reported the genomic regions for blanchability using QTL-seq approach on a RIL population [16].

SNP-trait association (STA) mapping of groundnut diverse panels has speed up the genomic region identification that are linked to agronomic traits by identifying ancestral or natural recombination events that led to non-random allele association at various loci throughout the genome [17, 18]. Compared to biparental linkage mapping, association mapping, allows greater mapping resolution [19]. Association mapping has acquired considerable pace in legumes, and there are several studies indicating markers associated with nutritional and agronomic traits [20]. With the availability of high-density SNP (Single Nucleotide Polymorphism) array and WGRS (Whole genome re-sequencing) genotyping data, there is much scope to study the economically important and other traits in groundnut, identify STAs using the sequencing based molecular markers, and deploying them in crop breeding programs [20–22]. Considering the above knowledge gap and available information, the present study aimed to identify STAs for economically important trait, blanchability, in the groundnut minicore collection [23].

In this study, a minicore collection comprising 184 diverse groundnut accessions was phenotyped for blanchability in two subsequent seasons; rainy (R) 2022 and post-rainy (PR) 2022–2023. We have conducted GWAS for the identification of significant SNP markers related to blanchability in groundnut, using three different models, viz., Bayesian-information and linkage-disequilibrium iteratively nested keyway (BLINK), Settlement of Unmappable Positions via Extensive Rearrangement (SUPER), and Fixed and random model Circulating Probability Unification (FarmCPU). These models enhance statistical power and computational efficiency, thus, improving the reliable selection of significant markers for breeding programs. Further, genes were identified from the LD region of associated significant STAs. Also, correlation and cluster analysis of blanchability with agronomic traits have identified genotypes with superior performance. These findings provide a clearer understanding of the trait, its potential governing regions, and underlying molecular mechanisms, thereby supporting the improvement of groundnut varieties through marker-assisted breeding.

## Materials and methods

### Plant material and experimental field design

The groundnut minicore collection of 184 accessions developed by Genebank of ICRISAT, Patancheru, was used in the experimental study [23]. It encompasses six botanical types viz., *hypogaea*, *hirsuta*, *fastigiata*, *peruviana*, *aequatoriana* and *vulgaris*, that collectively constitutes the genetic diversity present in the complete groundnut germplasm at ICRISAT. The experiment followed an alpha-lattice design across two seasons: rainy (2022) and post-rainy (2022–2023). Phenotyping was conducted in two replications per season at ICRISAT, Patancheru (Latitude 17°31′48″N; Longitude 78°16′12″E). The environmental conditions and climatic parameters for the field trials are mentioned in the Supplementary Table S1. Throughout both the seasons, standard recommended agronomical practices essential for groundnut cultivation were followed for healthy and good crop growth. Further, post-harvesting, the phenotypic observations were recorded for each replication. Additionally, the previously documented phenotyping data for various agronomical traits such as pods per plant (PPP), seed length (SDL), seed weight (SWT), days to maturity (DM), pod weight (PDWT), pod width (PDWD), and pod length (PLN), were also used for correlation and cluster analysis.

### Phenotypic evaluation for blanchability

**Fig. 1:**
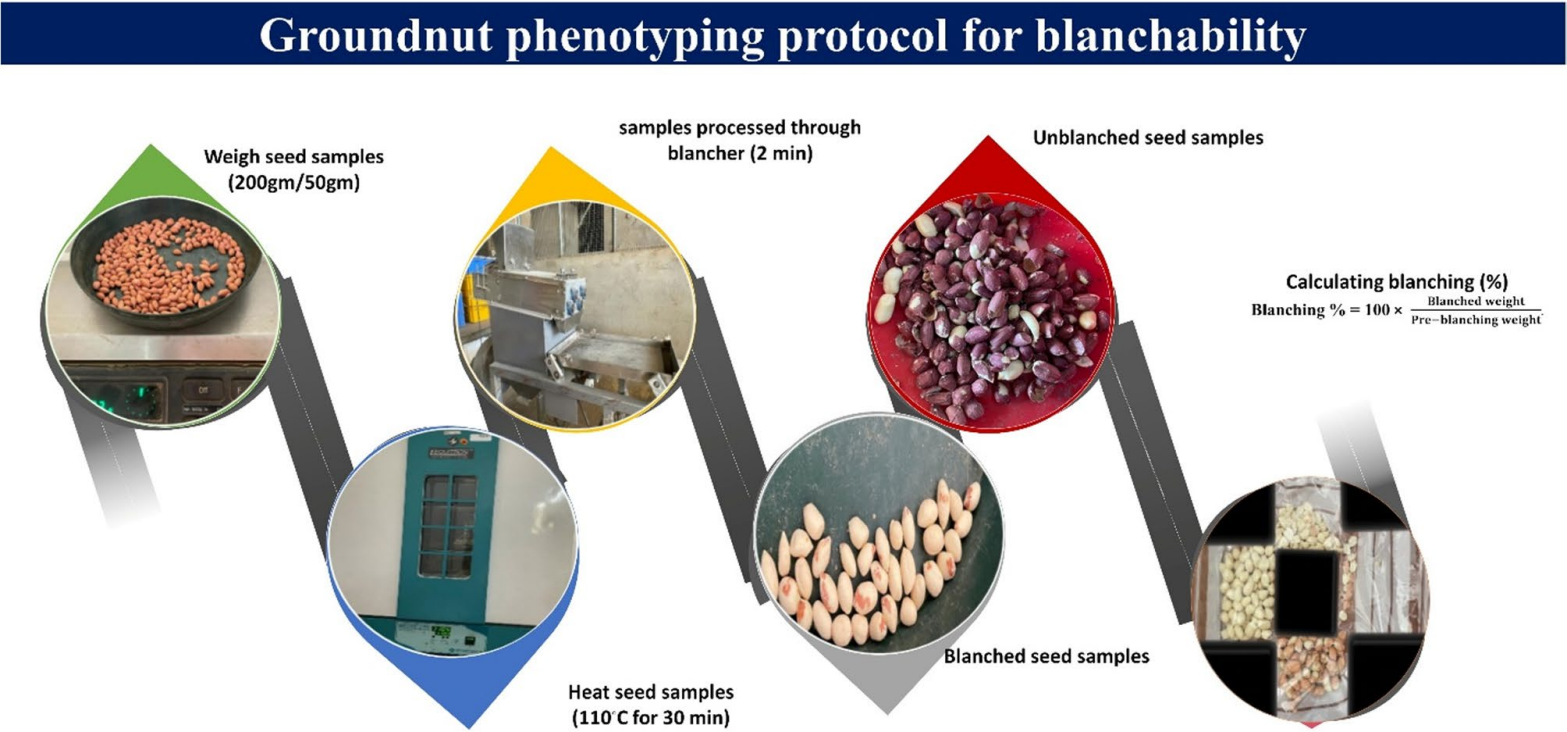
Protocol for phenotyping blanchability in groundnut

Phenotypic observations were taken on four sets of data: 200 gm (post-rainy), 50 gm (post-rainy), 50 gm (rainy), and 50 gm (average data of rainy and post-rainy). For assessment of groundnut blanchability, seeds were first graded (7–8 mm diameter, depending on genotype) to minimize variability in size and maturity. The pre-blanching weight of each genotype was recorded, followed by hot air oven treatment. As per the protocol [11], seed samples of ~ 200 gm and 50 gm were pre-heated at 110 °C for 30–35 min, reducing moisture content to 3.75–4.0%. Heated seeds were then cooled to room temperature for over 8 h before recording the data. Then, the samples were treated in a blancher at 30 rpm for 60 s (50 gm samples) and 120 s (200 g samples) [14] (Fig. 1). The weight of the blanched seeds as well as blanched splits was then observed and recorded, followed by calculating the blanching percentage, using the formula below:

$$\textbf{Blanchability}\ \% = \frac{\mathbf B\mathbf l\mathbf a\mathbf n\mathbf c\mathbf h\mathbf e\mathbf d\:\mathbf w\mathbf e\mathbf i\mathbf g\mathbf h\mathbf t}{\mathbf P\mathbf r\mathbf e-\mathbf b\mathbf l\mathbf a\mathbf n\mathbf c\mathbf h\mathbf i\mathbf n\mathbf g\:\mathbf w\mathbf e\mathbf i\mathbf g\mathbf h\mathbf t}\times\textbf{100}$$

### Phenotypic data analysis

The analysis of variance (ANOVA) and descriptive statistics was performed over the rainy (R) season of 2022 and post-rainy (PR) 2022–2023 using GenStat software version 15.0 (VSN International Ltd., Hemel Hempstead, UK) to assess phenotypic variation across seasons. In descriptive statistics, the Shapiro-wilk test was performed to test if the data follows normal distribution [24]. Differences between genotype replications in different seasons were evaluated using one-way ANOVA with the F-test at a 5% significance level. Similarly, two-way ANOVA was used to study the genotype and environment interaction. The ‘GGplot’ package in RStudio was used to generate frequency curves on histograms and boxplots to depict the distribution of phenotypic data among different agronomic types of groundnut. Pearson correlation was performed using the ‘Hmisc’ package, and the correlation heatmap was visualized with the ‘pheatmap’ package. Principal component analysis was performed to examine phenotypic variability in terms of blanchability, agronomic types, and other agronomic traits using 'FactoMineR' package.

### Identification of accessions as potential donors for blanchability

To identify desirable accessions from the minicore collection for stable blanchability and agronomic performance under diverse environments, the phenotypic data for blanchability along with days to maturity (DM), seed length (SDL) seed weight (SWT), pod weight (PDWT), pods per plant (PPP), pod width (PDWD), and pod length (PLN), were utilized for trait correlations and hierarchical clustering. Further, the promising accessions were identified based on favorable alleles.

### Genotyping with 58 K ‘Axiom_*Arachis*’ SNP array: A tool for high density genotyping in groundnut

A high-density assay of 58 K ‘Axiom_*Arachis*’ SNP Array [25] was used to generate genotypic information for 184 groundnut minicore accessions, and was utilized to find the STAs. Genomic DNA was extracted from tender leaves of 25–30 days old seedlings using Nucleospin Plant II kit (Macherey-Nagel, Düren, Germany). For genotyping, 20 ng/µl DNA from each sample was processed on the Affymetrix GeneTitan^®^ system with the Axiom_*Arachis*’ SNP array, comprising 58,233 SNP markers derived from resequencing of 41 wild diploid ancestors and tetraploid accessions [25, 26]. The 58 K SNPs represent an average of ~ 2,900 SNPs per chromosome across 20 chromosomes. Following SNP calling and data analysis conducted using Axiom™ Analysis Suite version 1.0 (Thermo Fisher Scientific, USA) to conduct quality control (QC) measures and choose samples that successfully cleared the QC test. TASSEL (Trait Analysis by Association Evolution and Linkage) v5.2.93 software [27] was used to extract the polymorphic markers to further analyse the data. Of the 58,233 SNPs for 184 groundnut minicore collection were retrieved from Axiom™ analysis suit, the derived SNPs were further filtered for removing low quality and rare SNPs with ≤ 5% minor allele frequency (MAF) and > 20% missing data. After stringent filtration, high-quality SNPs were subsequently employed for association mapping studies using GAPIT package in RStudio [28].

### Population structure analysis and genome-wide linkage disequilibrium

The population structure was estimated by PCA (principal component analysis), conducted using a genetic distance Roger’s matrix, to identify clusters of individual belonging to similar genetic background, which was calculated using selection tool package in RStudio. Squared Pearson’s correlation coefficients (r²) from pairwise SNP analysis were used to assess genome-wide linkage disequilibrium (LD) decay [29]. LD decay in the groundnut minicore collection panel was estimated using PopLDdecay v.3.29. and the SelectionTools package in RStudio.

### Genome-wide association analysis for identifying SNPs-associated with blanchability

SNP-trait association was carried out utilizing advanced GAPIT models, namely, BLINK, FarmCPU, and SUPER that employed both population structure (Q) and kinship (K) matrices. Quantile–Quantile (Q-Q) plots were generated using the CMplot package in RStudio by plotting observed –log10 *(p)* values against expected –log10 *(p)* values for all SNPs [30]. Manhattan plots were employed to depict chromosome-wise SNPs from marker–trait associations, plotting -log10(p) values of each SNP across the 20 chromosomes for blanchability, with corresponding season and sample size. The threshold to establish significant SNP-trait association was calculated by applying Bonferroni correction for *p*-value of 5.01 × 10^−6^ through negative log transformation of α/n [where, α refers to the overall significance level (0.05), n is the total number of SNPs employed for GWAS analysis (5044)]. The percentage of phenotypic variance explained (PVE) by each significant SNP was reported for all models. PVE was estimated as the squared correlation between SNP genotype and phenotype.

According to the distribution of SNPs, the threshold level of significance of associations between traits and SNPs was set at [–log 10 (*p*) < 10^−6^] for GWAS analysis by using SNP array that provided the best number of valid SNPs. SNP density plots, Q-Q plots, and Manhattan plots were created by the CMplot package of RStudio. Peanut base (https://Peanutbase.org/) was employed to detect the [31, 32] genes related to the STAs regions using the physical locations of significant SNPs in the reference genome sequence using GBrowse (cultivated peanut) version 1.

### Development and validation of kompetitive allele specific polymerase chain reaction (KASP) -based markers

The present association mapping study identified 58 SNPs with a significant association, of which nine highly significant markers were used for Kompetitive Allele-Specific PCR (KASP) assay development. These SNPs were selected from genomic locations near candidate genes on three distinct chromosomes and were redesigned into KASP markers using 50-bp upstream and downstream regions to create user-friendly and economical markers [33]. Two allele-specific forward primers and a generic reverse primer were designed for each SNP marker (Intertek Pvt. Ltd.). The KASP markers were validated across a panel of 25 high blanchability and low blanchability genotypes as well as ICRISAT breeding lines.

## Results

### Phenotypic analysis of blanchability

**Fig. 2:**
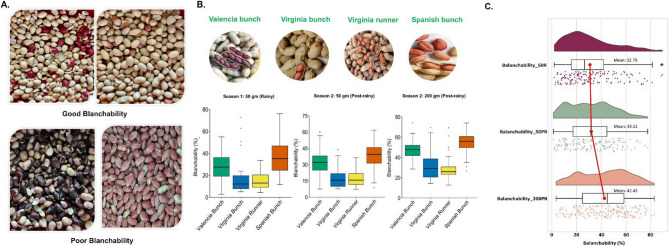
Different phenotypic analysis of blanchability in groundnut minicore collection: **A** Phenotypic variability for blanchability in groundnut minicore collection, **B **Box plot represents substantial variation in blanchability for the diverse agronomic types in groundnut minicore collection across different seasons and sample sizes, **C** The raincloud plot depicts the blanchability (%) values in season 1 (Blanchability_50R, pink color) and season 2 (Blanchability_50PR and 200PR, green and orange color; respectively), displaying considerable phenotypic variation with a continuous distribution of accession across both the seasons

Blanchability is an important economic trait, and its phenotypic variation is a key component in GWAS analysis. The range and mean phenotypic distribution in 184 accessions from the groundnut minicore collection across different seasons and sample size are presented in Fig. 2A, C. For season 2, with sample size 50gm, traits exhibit a symmetric distribution, on the contrary, for season 1, the distribution is slightly skewed to the right, indicating more values concentrated around 20 to 40% of blanchability, with fewer values beyond 60. Supplementary Table S2 shows the wide variability range in the blanchability in the minicore collection. This includes BLCB_50R: 92.63 (mean 32.79), BLCB_50PR: 96.76 (mean 33.11) and BLCB_200R: 94.52 (mean 42.43) (Supplementary Table S3). The trait and agronomic type wise phenotypic variation depicted that the Spanish Bunch and Virginia Runner possess the highest and lowest blanchability percentage; respectively **(**Fig. 2B**).**

Descriptive statistics showed that for the rainy (50 gm) and post-rainy (50 gm) seasons, values clustered around the mean with greater spread in the lower range, though the post-rainy mean was slightly higher. In contrast, post-rainy (200 gm) samples had markedly higher values, with a mean of ~ 42.57. Additionally, one-way and two-way ANOVA was performed. In one-way ANOVA, it was observed that for the rainy season and post-rainy season (50 gm), and post-rainy season (200 gm), the variation across genotypes was significant while the non-significant variation among replication suggests the accuracy of phenotypic data (Supplementary Table S4). In two-way ANOVA, the genotype and environment possess significant variation suggesting the effect of environment, while the genotype and sample size interaction is non-significant, that implies all genotypes respond similarly to sample size variations (Supplementary Table S5).

**Fig. 3:**
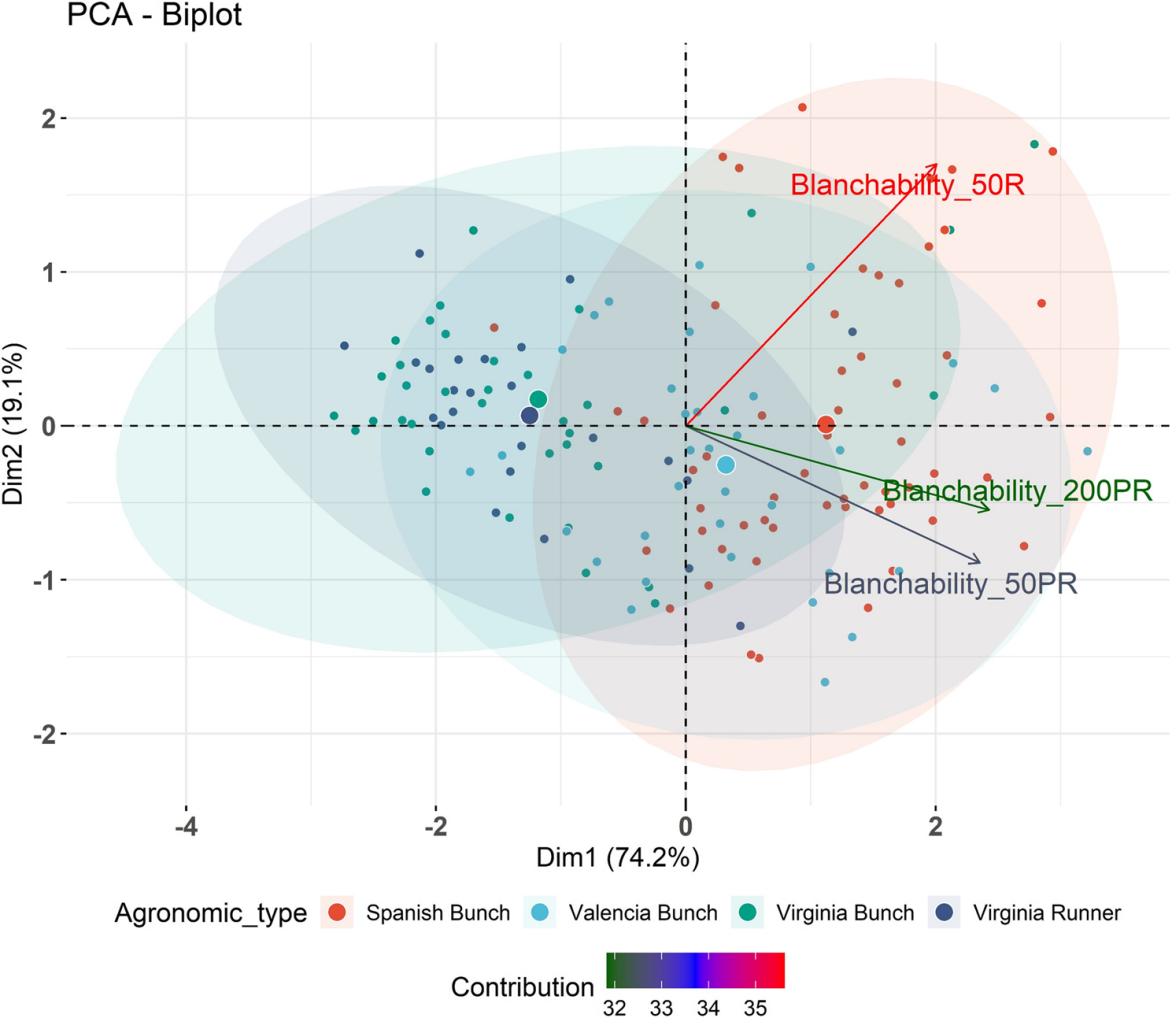
Principal Component Analysis (PCA) biplot depicting the relationship between blanchability traits and agronomic types in groundnut. The first two principal components (Dim1 and Dim2) explain 74.2% and 19.1% of the total variation, respectively. Vectors represent blanchability traits measured under different conditions (50R, 50PR, and 200PR), while individual genotypes are plotted and grouped according to their agronomic type (Spanish Bunch, Valencia Bunch, Virginia Bunch, and Virginia Runner). The variation has been observed more for blanchability in 50R. The ellipses indicate the distribution of genotypes within each agronomic group, and the color gradient reflects the relative contribution of variables to the principal components

**Fig. 4:**
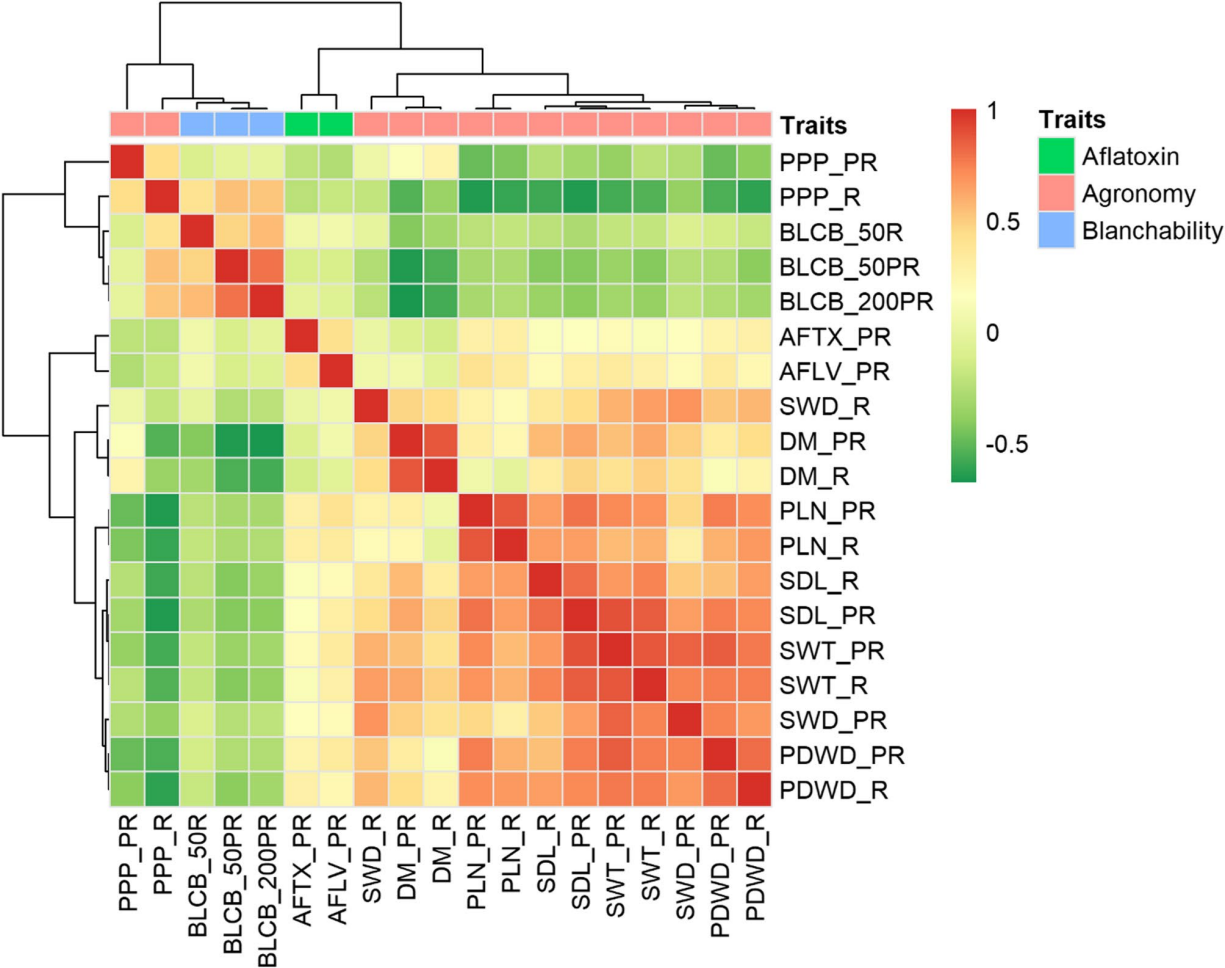
Pearson correlation analysis of blanchability, aflatoxin and agronomic traits evaluated across diverse environments post-rainy (PR) and rainy (R). The four traits including pods per plant (PPP), blanchability (BLCB), aflatoxin (AFTX), *A. flavus* infection (AFLV), days to maturity (DM), pod length (PLN), seed length (SDL), seed weight (SWT), seed width (SWD), and pod width (PDWD) were evaluated in rainy and post-rainy environment. The color reflect the strength of correlation and further cluster has been made. PPP (R) positively correlated with blanchability and hence clustered together and other agronomic traits and aflatoxin make separate clusters. Scale ranges from − 0.5 to 1, where the green color represents negative correlation while the red color depicts positive relationship between the traits

The PCA of the blanchability explained 93.3% of the total phenotypic variance (Fig. 3). The basis of clustering for accessions was not made on their agronomic type but had found to be varying degrees of relatedness, with Spanish Bunch and Valencia Bunch showing distinct separation, while Virginia Bunch and Virginia Runner overlap, indicating similarities. Blanchability_50PR and 200PR, were inversely related to the blanchability_50R. In addition, the PCA analysis of the blanchability and the agronomic traits showed that the first two PCs explained 62.3% of the total phenotypic variance. The analysis revealed notable trends between blanchability and agronomic traits. Aflatoxin (AFTX_PR) showed a negative correlation with pods per plant (PPP_PR), and blanchability (BLCB_200PR, 50PR, 50R) was negatively correlated with day to maturity (DM_R and DM_PR). Seed weight (SWT) was also significantly negatively correlated with blanchability (BLCB_200PR, 50PR). In contrast, aflatoxin (AFTX), *A. flavus* infection (AFLV) and other agronomic traits were positively correlated with blanchability (BLCB) across all environments, although these associations with AFTX and AFLV were not significant (Supplementary Figure S1). Pearson correlation analysis of blanchability, aflatoxin and agronomic traits showed that PPP (R) was positively correlated with blanchability and clustered with it whereas other agronomic traits and aflatoxin formed separate clusters (Supplementary Table S6 and S7) (Fig. 4).

### Potential donors for blanchability

Promising groundnut accessions with high productivity and blanchability can serve as donors for the breeding programs to be carried out to develop better varieties for fulfilling global demand. To identify the possible donors, we considered the correlations among the blanchability and agronomic characteristics, from various environments (Fig. 4). All the characteristics describe nearly 62.3% of total variation. The PCA factor plot revealed that PC1 and PC2 explained 46.3% and 16% of the variation in measured characteristics, respectively (Supplementary Figure S1).

Further, hierarchical cluster analysis classified the traits into three distinct clusters; Cluster1: days to maturity (DM_R, DM_PR); Cluster 2: seed width (SWD_PR, SWD_R), pod width (PDWD_PR, PDWD_R), pods per plant (PPP_R), seed length (SDL_PR, SDL_R); and Cluster 3: blanchability (BLCB_50R, BLCB_50PR, BLCB_200PR), seed weight (SWT_PR, SWT_R), pod length (PLN_PR, PLN_R), and pods per plant (PPP_PR) (Supplementary Figure S2). The top-performing accessions were identified within each cluster and compared among themselves, to identifying accessions for favorable combinations of two or more traits. Cluster1 comprised 21 accessions with higher days to maturity (Supplementary Table 8(a)). Cluster 2 contained eight accessions with higher seed width and pod width (Supplementary Table 8(b)). Cluster 3 included eight accessions with higher blanchability, seed weight, pod length and pods per plant (Supplementary Table 8(c)).

Based on cross-cluster comparisons, ICG297 (cluster 1) could be crossed with ICG15419, ICG11219, ICG332, ICG6022, ICG9809, ICG 4729 and ICG11687 (cluster 3) to breed large-seeded groundnut varieties with improved blanchability and seed weight. Similarly, ICG6766 (cluster 2) could be crossed with ICG332 (cluster 1) to combine longer maturity duration, with greater seed and pod width as well as pod length. The comparison between the clusters has revealed the common accessions; eleven accessions (ICG15419, ICG6022, ICG 11219, ICG4538, ICG11862, ICG6766, ICG9777, ICG4746, ICG2381, ICG8760 and ICG297), were common between cluster 2 and 3, ICG332 was common to cluster 1 and 3, and ICG14630 was common to cluster 1 and 2. Notably, ICG297 was present in all the three clusters, highlighting its potential as key donors for breeding programs aimed at enhancing, blanchability and agronomic traits in groundnut.

### Genotyping and SNP density

The 58 K ‘Axiom_*Arachis*’ SNP array was used for the genotyping of minicore collection. After stringent filtration, a total of 5044 high-quality SNPs were selected, across 167 accessions out of 184 as for some genotypes data was missing, this data was Subsequently utilized for GWAS using GAPIT package. For SNP array, SNP density across chromosomes, segmented into 1 Mb windows, certain chromosomes, such as Chr A02, Chr A03, Chr A04, and Chr B03, showed the highest overall SNP counts, with totals of 323, 293, 296 and 291 SNPs, respectively. In contrast, chromosomes Chr A10 and Chr B08 have relatively lower SNP counts; 198 and 206; respectively (Supplementary Figure S3).

### Population structure analysis and genome-wide linkage disequilibrium

**Fig. 5:**
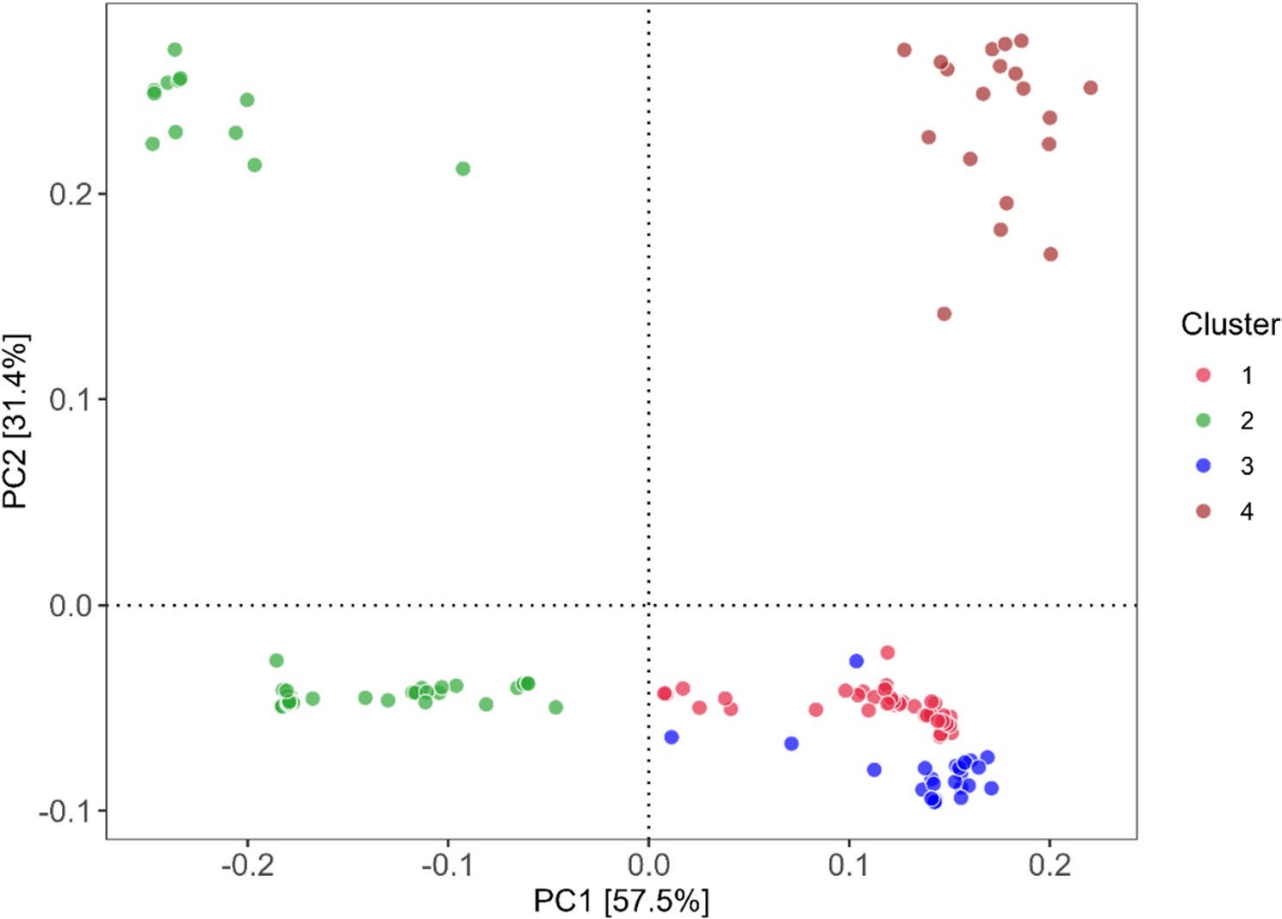
PCA depicting phenotypic variability of 88.9% for groundnut minicore collection, and 4 different clusters (pink, green, blue, brown) based on groundnut Sub-species, botanical variety and agronomic type. Cluster 1 (pink color): (46 genotypes): *fastigiata*, vulgaris, Spanish Bunch; Cluster 2 (green color): (75 genotypes): *hypogaea*, hypogaea, Virginia Bunch, Cluster 3 (Blue): (27 genotypes): *fastigiata*, fastigiata, Valencia Bunch, Cluster4 (Brown) (19 genotypes): *fastigiata*, fastigiata/vulgaris, Spanish Bunch, respectively

Heatmaps and dendograms of the kinship matrix indicated that there was clear clustering among the genotypes and was observed to be based on polymorphic SNPs (Fig. 5)(Supplementary Figure S4 (A) and (B)) (Supplementary Table S9). For SNP array, the population structure based on groundnut sub-species, botanical variety and agronomic type also revealed four distinct clusters: cluster 1 (46 genotypes): *fastigiata*, vulgaris, Spanish Bunch; cluster 2 (75 genotypes): *hypogaea*, hypogaea, Virginia Bunch; cluster 3 (27 genotypes): *fastigiata*, fastigiata, Valencia Bunch; cluster 4 (19 genotypes): *fastigiata*, fastigiata, vulgaris, Spanish Bunch, respectively (Supplementary Table S9). The graphical representation of the LD characteristics of the minicore collection is presented for SNP array data in Supplementary Figure S4 (B)**.** In the case of SNP array data, the mean r^2^ value for the genome was 0.12 and the LD decay was observed to start at a r^2^ value of 0.95 and half-decay at 0.15. The curve of LD decay cut off at 1Mbp which is the genome-wide critical distance to observe linkage. Therefore, markers linked to the same trait within this distance were classified as linked or associated. 

### Identifying SNPs and its associated candidate genes for blanchability

**Fig. 6:**
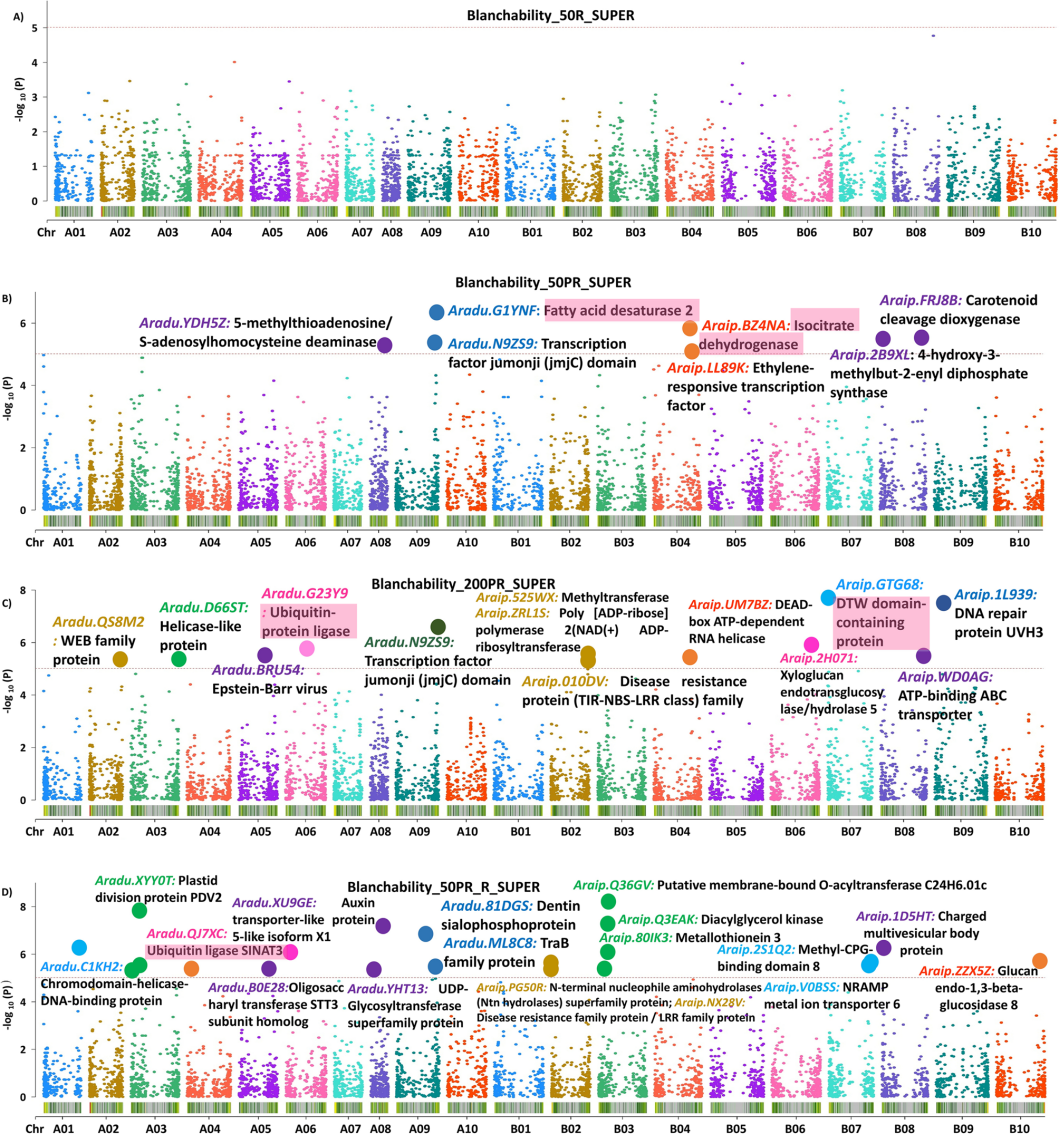
Manhattan plots representing the identified STAs associated with the blanchability on the basis SUPER model in GAPIT for A: Blanchability 50R (50 gm sample size in rainy season), B: Blanchability 50PR (50 gm sample size in post-rainy season), C: Blanchability 200PR (200 gm sample size in post-rainy season) and D: Blanchability 50PR_R (50 gm sample size in post-rainy and rainy season), respectively. In these Manhattan plots, the highlighted (pink) region shows the gene associated with the validated polymorphic markers on chromosome A01, A06, B04 and B07; respectively. The other dots represent the significant STAs, and the genes found to be associated with blanchability. X-axis represents the chromosome number while the Y-axis represents -log_10_ (*p*) values of the SNPs detected.

GWAS analysis was conducted based on high-density filtered 5044 SNPs (< 20% missing data) distributed across the 20 groundnut chromosomes. The threshold level of -log10 *p* value was fixed at 5.0 for SNP array analysis above which the SNPs are reported to be significantly associated (Fig. 6, Supplementary Figure S5, S6). GWAS results revealed total of 58 highly significant STAs for blanchability (Supplementary Table S10). Of the 58 STAs, 21 were identified for 200PR with -log10 *p* values ranging from 1.61 × 10^−8^ to 6.28 × 10^−6^ which explained phenotypic variance (PVE) as high as 35.28%, and 28 STAs from 50_PR_R mean data set, with -log10 *p* values ranging from 5.45 × 10^−13^ to 9.36 × 10^−6^ explained PVE as high as 39.03% of PVE, further from 50R, 4 STAs were identified with -log10 ‘*p*’ values ranging from 1.18 × 10^−8^ to 1.18 × 10^−6^, and PVE as high as 45.93% while for 50PR, 7 STAs with -log10 ‘*p*’ values ranging from 3.79 × 10^−7^ to 8.80 × 10^−6^ and PVE as high as 32.24% were found. Finally, the nine SNPs that were identified for blanchability from the analysis with the 4 different data sets, and these SNPs were located on chromosome A01 (1), A05 (2), A06 (1), A09 (2), B04 (2), and B07 (1). Among these SNPs, B07_2964357 (AX_147254562) was observed to possess the highest value for phenotypic variation of 35.28% with a -log10 *p* value of 3.69 × 10^−6^ (Table 1) (Figure 6, Supplementary Figure S5, S6).

**Table 1:** Marker trait association identified candidate genes and significant STAs for groundnut blanchability using 58 K “Axiom_*Arachis*” array

Blanchability	Markers	SNP ID	Chr	Position	*p* value	Model	SNP effect	PVE (%)	Gene ID	Annotations
BLCB_50R, BLCB_50_PR_R	B07_118262617	AX_147256471	B07	118,262,617	1.05E-07	a, c	13.72	24.87	*Araip.V0BSS*	*NRAMP metal ion transporter 6*
BLCB_50PR	A09_114692575	AX_147234396	A09	114,692,575	4.28E-06	c	11.99	5.08	*Aradu.G1YNF*	*fatty acid desaturase 2*
BLCB_50PR	B04_105437967	AX_147247942	B04	105,437,967	1.40E-06	c	−12.43	6.10	*Araip.LL89K*	*ethylene-responsive transcription factor*
BLCB_50PR	B04_108209828	AX_147247969	B04	108,209,828	8.80E-06	c	11.97	5.32	*Araip.BZ4NA*	*isocitrate dehydrogenase*
BLCB_50PR, BLCB_200PR	A09_115432582	AX_147234469	A09	115,432,582	3.79E-07	c	12.44	32.24	*Aradu.N9ZS9*	*Transcription factor jumonji (jmjC) domain-containing protein*
BLCB_50PR	A08_41497800	AX_147231354	A08	41,497,800	6.13E-06	c	−21.86	1.68	*Aradu.YDH5Z*	*5-methylthioadenosine/S-adenosylhomocysteine deaminase*
BLCB_50PR	B08_3609752	AX_147257272	B08	3,609,752	3.01E-06	c	−21.88	17.13	*Araip.2B9XL*	*4-hydroxy-3-methylbut-2-enyl diphosphate synthase*
BLCB_50PR	B08_110518175	AX_147258765	B08	110,518,175	3.01E-06	c	−21.88	14.18	*Araip.FRJ8B*	*carotenoid cleavage dioxygenase 1*
BLCB_200PR	A03_44353475	AX_147216911	A03	44,353,475	2.14E-07	a, b, c	7.36	20.00	*Aradu.D66ST*	*Helicase-like protein*
BLCB_200PR	B07_2964357	AX_147254562	B07	2,964,357	3.69E-06	a, b, c	−9.66	35.29	*Araip.GTG68*	*DTW domain-containing protein*
BLCB_200PR	B09_26207513	AX_147260814	B09	26,207,513	2.30E-07	a, b, c	8.26	17.59	*Araip.1L939*	*DNA repair protein UVH3*
BLCB_200PR	A05_71291610	AX_147222611	A05	71,291,610	2.95E-06	c	10.37	0.41	*Aradu.BRU54*	*Epstein-Barr virus EBNA-1*
BLCB_200PR	A06_57729112	AX_147225399	A06	57,729,112	1.89E-06	c	10.96	1	*Aradu.G23Y9*	*ubiquitin-protein ligase 7*
BLCB_200PR	A02_84589354	AX_147214749	A02	84,589,354	4.41E-06	c	−7.43	0.91	*Aradu.QS8M2*	*WEB family protein*
BLCB_200PR	A03_133198789	AX_147218592	A03	133,198,789	4.94E-06	c	−7.26	3.45	*Aradu.4PJ68*	*unknown protein*
BLCB_200PR	B04_101149286	AX_147247867	B04	101,149,286	4.36E-06	c	7.28	9.74	*Araip.UM7BZ*	*DEAD-box ATP-dependent RNA helicase*
BLCB_200PR	B06_114586674	AX_147253251	B06	114,586,674	1.12E-06	c	16.11	32.81	*Araip.2H071*	*xyloglucan endotransglucosylase/hydrolase 5*
BLCB_50_PR_R	A01_230616	AX_147207637	A01	230,616	4.37E-06	a	6.65	5.82	*Aradu.0B4KA*	*uncharacterized protein*
BLCB_50_PR_R	A03_29722463	AX_147216570	A03	29,722,463	5.45E-13	b, c	−9.48	25.49	*Aradu.K3EGK*	*Unknown protein*
BLCB_50_PR_R	A07_12521563	AX_147227734	A07	12,521,563	2.91E-06	b	−6.16	17.60	*Aradu.V4VHC*	*pumilio 2*
BLCB_50_PR_R	A05_85198860	AX_147222844	A05	85,198,860	5.08E-06	a, b, c	−6.83	23.26	*Aradu.B0E28*	*Oligosaccharyl transferase STT3 subunit homolog*
BLCB_50_PR_R	A01_97179228	AX_147211857	A01	97,179,228	5.36E-07	c	12.42	1.46	*Aradu.C1KH2*	*chromodomain-helicase-DNA-binding protein*
BLCB_50_PR_R	A04_10026356	AX_147219449	A04	10,026,356	4.46E-06	c	11.77	7.05	*Aradu.MI7GZ*	*Unknown protein*
BLCB_50_PR_R	A06_12625218	AX_147224932	A06	12,625,218	7.29E-07	c	6.03	7.12	*Aradu.QJ7XC*	*ubiquitin ligase SINAT3*
BLCB_50_PR_R	B02_163783	AX_147239773	B02	163,783	4.85E-06	c	11.41	2.12	*Araip.PG50R*	*N-terminal nucleophile aminohydrolases (Ntn hydrolases) superfamily protein*
BLCB_50_PR_R	B03_25025384	AX_147244167	B03	25,025,384	4.23E-06	c	11.16	4.00	*Araip.Q36GV*	*putative membrane-bound O-acyltransferase C24H6.01c*
BLCB_50_PR_R	B03_31216966	AX_147244335	B03	31,216,966	5.94E-09	c	−8.19	13	*Araip.80IK3*	*metallothionein 3*
BLCB_50_PR_R	B08_1018858	AX_147256997	B08	1,018,858	6.85E-07	c	−6.49	1.24	*Araip.1D5HT*	*charged multivesicular body protein*

Legends: - a: FarmCPU, b: BLINK, c: SUPER, BLCB_50R (Blanchability for 50 gm rainy sample), BLCB_50PR (Blanchability for 50 gm post-rainy sample), BLCB_200PR (Blanchability for 200 gm post-rainy sample), BLCB_50_PR_R (Blanchability for mean of 50 gm sample rainy and post-rainy). Some SNPs are positively regulating the trait (SNP effect positive) while some shows negative regulation (SNP effect negative)

A total number of nine SNPs were identified to possess genes that were associated with seedcoat and cell wall adhesion property **(**Table 1**)** viz., *Oligosaccharyl transferase STT3 subunit homolog (Aradu.B0E28)*,* NRAMP metal ion transporter 6 (Araip.V0BSS); Transcription factor jumonji (jmjC) domain-containing protein (Aradu.N9ZS9); 5-methylthioadenosine/S-adenosylhomocysteine deaminase (Aradu.YDH5Z)*,* fatty acid desaturase 2 (Aradu.G1YNF)*,* ethylene-responsive transcription factor 7 (Araip.LL89K)*,* isocitrate dehydrogenase (Araip.BZ4NA); 4-hydroxy-3-methylbut-2-enyl diphosphate synthase (Araip.2B9XL); xyloglucan endotransglucosylase/hydrolase 5 (Araip.2H071); putative membrane-bound O-acyltransferase (Araip.Q36GV)*,* ubiquitin ligase SINAT3 (Aradu.G23Y9); N-terminal nucleophile aminohydrolases (Ntn hydrolases) superfamily protein (Araip.PG50R);* and *charged multivesicular body protein (Araip.1D5HT)*.

### Allelic effects of stable STAs and validation of blanchability SNPs

A total of nine significant STAs were observed to be distinguishing for the high and low blanchability genotypes (Supplementary Figure S7). All the nine SNP markers A01_230616 (50_PR_R), A05_71291610 (50_PR_R,200PR), A05_85198860 (50_PR_R), A06_57729112 (50_PR_R,200PR), A09_114692575 (50_PR_R,50PR), A09_115432582 (50_PR_R,200PR), B04_105437967 (50_PR_R,200PR,50PR), B04_108209828 (50_PR_R,50PR), and B07_2964357 (50_PR_R,200PR), showed stable STAs with blanchability trait and were further allowed to be used for determining the individual allelic effects on the studied traits. The identified alleles of these nine SNP markers were observed to have substantial effects on blanchability (Supplementary Figure S8).

### KASP markers development and validation for blanchability

**Fig. 7:**
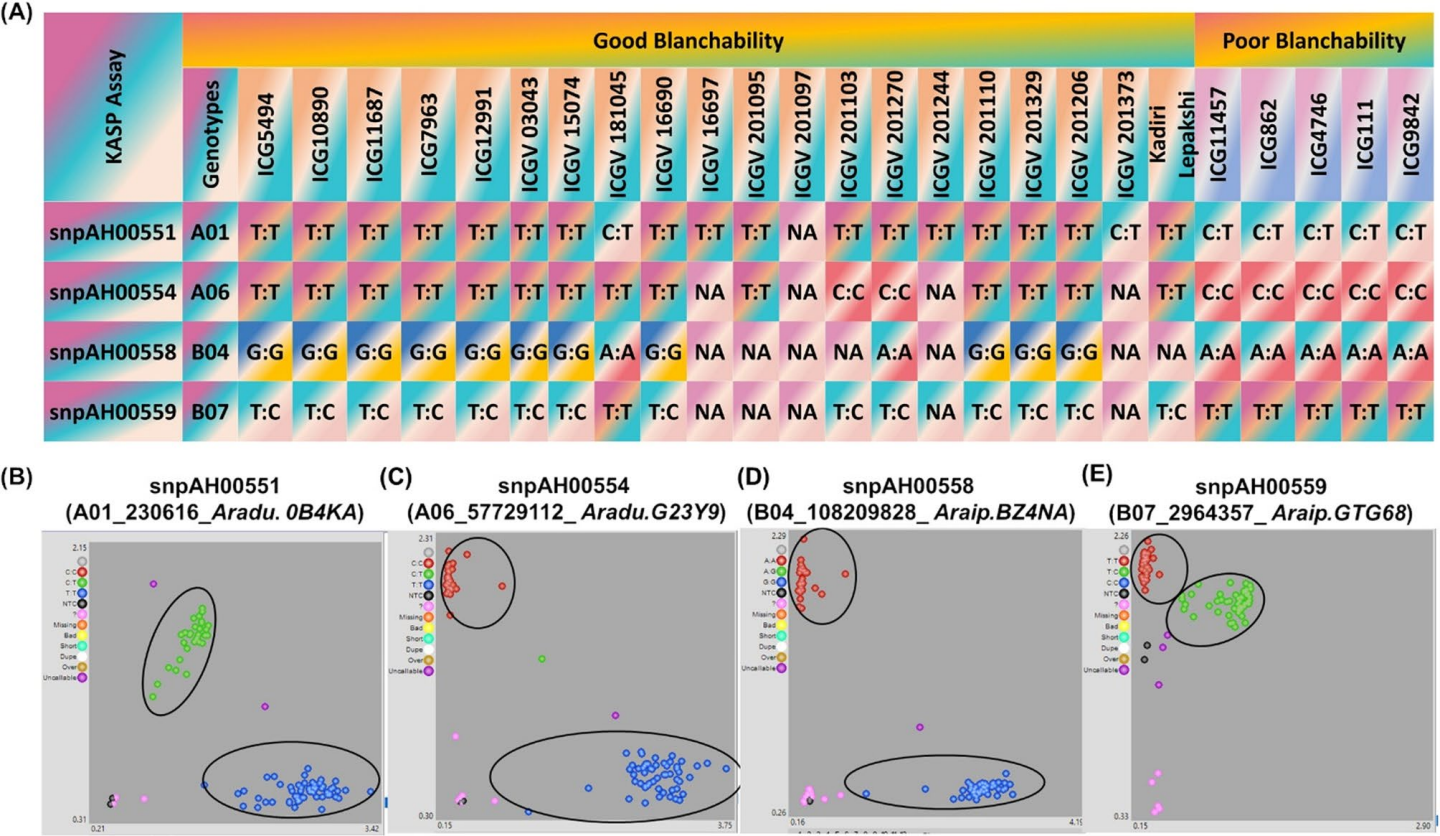
Development and validation of Kompetitive allele specific polymerase chain reaction (KASP) markers from potential candidate genes identified for blanchability.** A** Validation panel includes 20 high blanchability genotypes and groundnut varieties (50–80%-blanchability), and five low blanchability genotypes (1–10%-blanchability). Four single-nucleotide polymorphisms (SNPs) show clear homozygous clusters and heterozygous cluster for (**B**) snpAH00551 (A01_230616) (*AhBL01*) for uncharacterized protein (*Aradu.0B4KA*) gene, **C** snpAH00554 (A06_57729112) (*AhBL02*) for *ubiquitin-protein ligase* (*Aradu.G23Y9*) gene (**D**) snpAH00558 (B04_108209828) (*AhBL03*) for *isocitrate dehydrogenase* (*Araip.BZ4NA*) gene, **E** snpAH00559 (B07_2964357)(*AhBL04*) for *DTW domain-containing protein* (*Araip.GTG68*) gene

KASP markers represent cost-effective and efficient genotyping tools that enables early-generation selection among breeding lines, allowing indirect selection for specific phenotypes [34]. For the development and validation of the diagnostic markers for blanchability, nine SNPs were selected. Six of these were located on chromosomes A05, A09, and B04 (two SNPs each), while the remaining three were distributed across A01, A06, and B07 (one SNP each). These SNPS were substantially targeted for KASP markers development, and primers were Successfully designed and developed for 9 SNPs; A01_230616 (TT/CC), A05_71291610 (CC/TT), A05_85198860 (TT/GG), A06_57729112 (TT/CC), A09_115432582 (GG/AA), B04_105437967 (CC/TT), A09_114692575 (GG/AA), B04_108209828 (GG/AA) and B07_2964357 (CC/TT). These developed markers were validated on a validation panel with contrasting blanchability. The validation panel comprised of high and low blanchability genotypes and breeding lines ranging from 6 to 80%. Of the nine KASP markers selected for validation, four KASPs (snpAH00551, snpAH00554, snpAH00558 and snpAH00559) showed polymorphism in the designed validation panel and were clearly differentiating between genotypes that possess high and low blanchability. These KASP markers were localized on genomic region detected on chromosome A01, A06, B04 and B07 (Fig. 7). Therefore, the validated highly polymorphic KASP markers for high and low blanchability genotypes and can be used as potential diagnostic markers for very early-stage selection of blanchability breeding material at the varietal development process.

## Discussion

Groundnut is a globally cultivated food legume, celebrated for its high protein and unsaturated oil content. Blanchability quality becomes a significant trait regarding the development and utility of groundnut breeding lines for edible consumption [35]. In the research conducted so far for the blanchability trait, runner-type groundnut has been predominantly utilized, and several laboratory-scale blanchers have been customized to support breeding programs in identifying high-blanchability genotypes [36–39]. In the present study, a minicore collection panel was analysed and multi-environment phenotyping was carried out for the identification of the genomic regions and candidate genes that were associated with blanchability. This approach aims to enhance the understanding of the genetic factors influencing this economically important trait in groundnut cultivation.

In addition, some accessions identified through cluster analysis can serve as potential donors and would further facilitate for the development of breeding lines with improved traits. These accessions would be harboring beneficial alleles governing blanchability along with agronomical traits in groundnut. Accession ICG297 (cluster 1) could be crossed with ICG15419, ICG11219, ICG332, ICG6022, ICG9809, ICG 4729 and ICG11687 (cluster 3) to breed large-seeded groundnut varieties with high blanchability and seed weight. ICG6766 (cluster 2) could be crossed with ICG332 (cluster 1) to breed high days to maturity, seed width, pod width and pod length varieties. Accession (ICG297) common among all three, 1, 2 and 3, combine blanchability and agronomic traits, that could be effectively used in breeding programs as potential donor.

The cultivated agronomic types of groundnut include, ‘Spanish Bunch’, ‘Valencia Bunch’, ‘Virginia Runner’, and ‘Virginia Bunch’. These types exhibit distinct phenotypic traits such as branching habit, seed size, and pod size, reflecting their adaptation to specific production environments. In the present study, Spanish Bunch types exhibited high blanchability, whereas most of the Virginia Runner types showed low blanchability. As per the industrial demand, to produce groundnut processed food such as groundnut butter, cookies, and chocolates, industries can prefer Spanish Bunch agronomic types (high blanchability), while for beer nuts and other seed based confectionary products Virginia Runner can be considered (low blanchability).

Genomics-assisted breeding has been used widely for improvisation of several significantly important traits such as disease resistance, nutritional content, abiotic stress resistance in groundnut [40–42]. GWAS has become the primary method for identifying STAs related to complex traits of interest. Utilizing advanced models like FarmCPU, BLINK and SUPER significantly enhances the reliability and precision of identifying genetic associations in plant genomics [43–45]. Resequencing-based genotyping reveals extensive natural variations, allowing for the exploration of functional genes and elite alleles within natural populations through GWAS. This approach enables us to identify valuable genetic traits that can enhance breeding programs and improve crop performance [46, 47]. In this study, we identified a total of 58 significant STAs (Supplementary Table S10) for the blanchability trait using 58 K ‘Axiom_*Arachis*’ array; through GWAS using a minicore collection panel. Among which four SNP markers were validated, that were distinguishing the high (50–80%) and low blanchability lines (1–30%). These markers are snpAH00551 (A01), snpAH00554 (A06), snpAH00558 (B04) and snpAH00559 (B07).

These results are supported by the previous study reporting the same chromosome to be associated with blanchability; however, the genomic region varies [16]. On chromosome A06, associated SNPs from *Arahy.06_108*,*665*,*514* and *Arahy.06_108*,*812*,*907* have been reported earlier, positioned distantly from the polymorphic SNP *Aradu.06_57*,*729*,*112* identified and validated in our study. This underscores A06 as an important genomic region for blanchability. Additionally, the markers from chromosome A06 (snpAH00181) and B01(snpAH00187) have also been validated by the Peanut Company of Australia Pty Ltd, A Bega Company, Australia, University of Georgia (UGA), USA and ICRISAT, India.

Moreover, groundnut blanchability, is a trait influenced by genetic and environmental factors. Studies have associated blanchability with specific genomic regions or quantitative trait loci (QTLs). Previous research suggests that QTLs linked to groundnut blanchability are often identified on chromosome A06 and B01, these associations are determined through performing QTL-seq with two bulks of breeding lines from three populations. In addition, the KASP markers were developed from the most important SNPs from the QTLs on A06 *(Arahy.06_108*,*665*,*514*,* Arahy.06 _108*,*812*,*907) and B01 (Arahy.11_15*,*264*,*657 Arahy.11_16*,*329*,*544*,* Arahy.11_18*,*994*,*278*) [16]. The linkage-drag which is reported to be associated with two prominent *A. cardenasii* introgressions (A02 and A03) have been conferred for disease resistance and negatively affects blanchability. As a result, when breeding for disease resistance using wild germplasm, there is a risk of co-introduction of undesirable traits such as reduced blanchability due to their close genetic linkage [16]. However, if there is a requirement of skin for processing application, then this linkage-drag could be proven to be beneficial such as in case of beer nut and other seed based confectionary products. As it would enable to select disease resistant and blanching resistant varieties.

Blanchability has received limited attention until recently; however, efforts are now underway to identify STAs linked to high blanchability in groundnut. The objective is to develop parental lines with improved blanchability that can serve as donors in breeding programs for groundnut improvement. The SNP allele analysis of the diverse minicore collection accessions for the identified significant STAs indicated that accessions carrying all favorable blanchability alleles tend to exhibit good blanchability. The validation panel for blanchability includes extreme genotypes representing high blanchability and low blanchability genotypes, along with breeding lines. Those STAs which distinguish clearly high and low blanchability lines hold promise for developing molecular markers, and accessions with all favorable high blanchability alleles can serve as donors in marker assisted backcrossing (MABC) programs aimed at achieving high blanchability lines. Among these, four markers snpAH00551 (A01), snpAH00554 (A06), snpAH00558 (B04) and snpAH00559 (B07) were successfully validated across minicore collection and groundnut breeding lines.

Groundnut genome sequencing accounted for genome size of 1.2 Gb in *A. duranensis* (36,734 genes) and for *A. ipaensis* it was 1.5 Gb (41,840 genes) and demystified the role of several genes for blanchability. Physical locations of each SNP marker of the current study were compared with the groundnut genome sequence to identify the gene function that underlies the corresponding SNP using Peanut base (https://Peanutbase.org/). Based on the previously reported genes documented in the literature, candidate genes which were previously reported to control seed coat integrity and adhesion were subsequently found to be candidate genes controlling blanchability.

The polymorphic SNPs, snpAH00551 (A01,* AhBL01*), snpAH00554 (A06, *AhBL02*), snpAH00558 (B04, *AhBL03*) and snpAH00559 (B07, *AhBL04*) identified in the validation analysis are linked to the *genes isocitrate dehydrogenase* (*Araip.BZ4NA*) (B04, *AhBL03*) and *ubiquitin protein-ligase* (*Aradu.G23Y9,AhBL02*) (A06). These genes influence cell wall dynamics, thereby affecting the ease of seed coat removal, a critical factor in groundnut blanchability. *Isocitrate dehydrogenase* generates α-ketoglutarate (α-KG), which regulates cell wall–loosening enzymes such as *pectin methylesterases* and *expansins*. Alterations in seed coat integrity modify adhesion properties, directly impacting blanchability [48].

*Ubiquitin protein-ligase* and *ubiquitin ligase SINAT3* (a RING-type ubiquitin ligase) regulates protein degradation via the ubiquitin-proteasome system (UPS), affecting stress response, hormonal signalling, and cell wall modifications. Moreover, *SINAT3* is involved in abiotic stress response pathways, including oxidative stress and hormone signalling (e.g., auxin, ethylene), which govern seed coat integrity and blanchability by influencing pectin breakdown and adhesion strength [49]. It likely interacts with transcription factors (e.g., NAC, MYB) involved in cell wall degradation, modulating lignin biosynthesis and pectin remodelling [50].

The interplay between oxidative metabolism (*Isocitrate dehydrogenase*) and protein turnover (*Ubiquitin protein-ligase* and *Ubiquitin ligase SINAT3*) determines seed coat adhesion strength, ultimately influencing blanchability. *Isocitrate dehydrogenase* contributes to blanchability by regulating α-KG-dependent enzymes involved in seed coat loosening. On the other hand, *Ubiquitin protein-ligase* and *Ubiquitin ligase SINAT3* impacts blanchability by targeting proteins that control seed coat adhesion and degradation. The pathway can be further validated by transcriptomic and proteomic analysis of high- and low-blanchability groundnut genotypes to assess differential expression of *ubiquitin protein-ligase/ubiquitin ligase SINAT3*, *isocitrate dehydrogenase* and cell-wall-modifying enzymes. Furthermore, if *isocitrate dehydrogenase* activity is high, lignin/pectin biosynthesis is enhanced, leading to a stronger seed coat and lower blanchability. If *SINAT3* degrades key regulators of cell wall biosynthesis, this can lead to a weaker seed coat and higher blanchability. *Isocitrate dehydrogenase* contributes to blanchability by regulating metabolic flux and supplying intermediates for seed coat biosynthesis, while *SINAT3* acts through the ubiquitin–proteasome system to modulate stress responses and cell wall composition. Their combined effects, mediated by oxidative stress pathways, hormonal signaling, and protein degradation, ultimately influence seed coat adhesion and blanchability in groundnut.

Additionally, we have determined genes that are associated to cell wall biosynthesis, modifications, remodeling and adhesion proteins such as *xyloglucan endotransglucosylase*, and *oligosaccharyl transferase STT3*,* putative membrane-bound O-acyltransferase* that influence seed coat’s biochemical properties. For instance, x*yloglucan endotransglucosylase (XETs)*, participated in cell wall modification and maintained cell wall plasticity, helping plant in tolerating stress such as salinity stress [51]. *XETs* modify xyloglucans, the major hemicellulosic components of the plant cell wall. They play a pivotal role in restructuring the cell wall by cleaving and rejoining xyloglucan chains, facilitating cell wall loosening and expansion. This process is essential for preserving cell wall integrity, regulating cell–cell adhesion, and supporting plant growth and development. During seed coat formation, *XET* activity is particularly important, as precise cell wall modifications are required for proper development [52].

*STT3* functions as a core component of the *oligosaccharyltransferase (OST)* complex, which mediates N-linked glycosylation. Glycoproteins localized in the cell wall or membrane can influence cell wall remodeling and stability, mutation in *stt3a* disturbs these processes, impairing essential adaptation mechanisms [53]. Also, *STT3* is a catalytic subunit of the *OST* complex, responsible for transferring oligosaccharides to nascent proteins during N-linked glycosylation. Proper glycosylation is essential for the stability and function of many cell wall proteins, thereby influencing cell wall integrity and cell-cell adhesion. The structure of the seed coat is tightly regulated during seed maturation, and fatty acids, synthesized and modified by *Fatty acid desaturases (FADs). FADs*, are part of the cuticle (a waxy protective layer) that forms on the outer surface. In seed coats, the fatty acid profile affects the permeability and protective functions of the seed coat, thereby influencing seed development and viability [54]. It plays a vital role for determining the lipid composition of the seed coat, affecting its physical properties and functions, including protection, permeability, and regulation of dormancy and germination.

All these enzymes are integral to the dynamic processes of cell wall construction, modification, and maintenance, directly affecting seed coat development, cell wall integrity, and adhesion in plants. By modifying interactions within the cell wall, these genes may influence how tightly the seed coat adheres to the cotyledons. Strong adhesion can make blanching more difficult, while effective modification might facilitate easier removal of the seed coat.

## Conclusion

Blanchability is a trait of tremendous economic importance as there is a significant energy, time and cost requirement to remove the groundnut seedcoats from low blanching varieties. Product processing ultimately becomes cumbersome leading to re-processing. This ultimately leads to considerable loss of kernels during the processing and sorting of the groundnut. The present study utilized genotypic data from 58 K ‘Axiom_*Arachis*’ array and phenotypic data collected across two seasons from a diverse minicore panel to perform a genome-wide association study. The phenotypic analysis reveals that the Spanish Bunch agronomic types are possessed to have high blanchability, additionally, the statistical analysis has revealed the effect of sample size and environment on blanchability trait. ICG297 genotype has been identified to be the superior potential donor for blanchability along with other agronomic traits. Further, the GWAS analysis has identified significant STAs associated with potential candidate genes such as *isocitrate dehydrogenase* and *ubiquitin ligase protein* related to blanchability, highlighting the critical role of these genes in the dynamic processes of cell wall construction, modification, and maintenance which have a direct impact on the development of seed coats, the integrity of cell walls, and plant adhesion. Ultimately, four KASP markers snpAH00551 (A01, *AhBL01*), snpAH00554 (A06, *AhBL02*), snpAH00558 (B04, *AhBL03*) and snpAH00559 (B07, *AhBL04*) were validated from 58 K ‘Axiom_*Arachis*’ array exhibiting clear polymorphism between high and low blanchability genotypes, which could be incorporated in breeding programs. Furthermore, conducting haplotype analysis on these candidate genes could facilitate the identification of superior haplotypes for blanchability, which can be leveraged in haplotype-based breeding strategies.

## Supplementary Information

Supplementary material 1.

Supplementary material 2.

## Data Availability

The phenotypic data used in this work is provided in Supplementary Table S2. The sequencing data generated in this study is deposited in NCBI with bio-project ID PRJNA1002116.

## Abbreviations

GWAS: Genome-wide association studies
STAs: SNP-trait associations
MUFA: Mono-unsaturated fatty acid
PR: Post-rainy
R: Rainy
PVE: Phenotypic variance explained
SNP: Single nucleotide polymorphism
PPP: Pods per plant
SDL: Seed length
SWT: Seed weight
PDWT: Pod weight
PDWD: Pod width
PLN: Pod length
DM: Days to maturity
BLCB: Blanchability
AFTX: Aflatoxin
KASP: Kompetitive Allele-Specific PCR
MAF: Minor allele frequency
XETs: Xyloglucan endotransglucosylase
OST: Oligosaccharyl transferase
FADs: Fatty acid desaturases
GAB: Genomic-assisted breeding
Q-Q: Quantile-Quantile
MABC: Marker assisted backcrossing
