# Supplementary material for: Identification of high blanchability donors, candidate genes and markers in groundnut
Source: BMC Plant Biol. 2025 Oct 21;25:1409. doi: 10.1186/s12870-025-07309-9 (PMC12539096; doi:10.1186/s12870-025-07309-9)
Supplement: Supplementary file 2 — Supplementary material 2. [file 12870_2025_7309_MOESM2_ESM.docx]

**Identification of High Blanchability Donors, Candidate genes and Markers in Groundnut**

Priya Shah^1,2^, Sunil S. Gangurde^1,3^, Ramachandran Senthil^1^, Prashant Singam^2^, Ovais Hamid Peerzada^1^, Pasupuleti Janila^1^, Kuldeep Singh^1^, Sean Mayes^1^, Manish K. Pandey^1^*

^1^Center for Pre-Breeding Research (CPBR) and Center of Excellence in Genomics & Systems Biology (CEGSB), International Crops Research Institute for the Semi-Arid Tropics (ICRISAT), Hyderabad-502324, Telangana, India

^2^Department of Genetics, Osmania University, Hyderabad-500007, Telangana, India

^3^CIMMYT-China International Maize and Wheat Research Center, Shandong Agricultural University, Taian-271000, Shandong, China

***Correspondence:** [manish.pandey@icrisat.org](mailto:manish.pandey@icrisat.org)

**Supplementary Figures.**


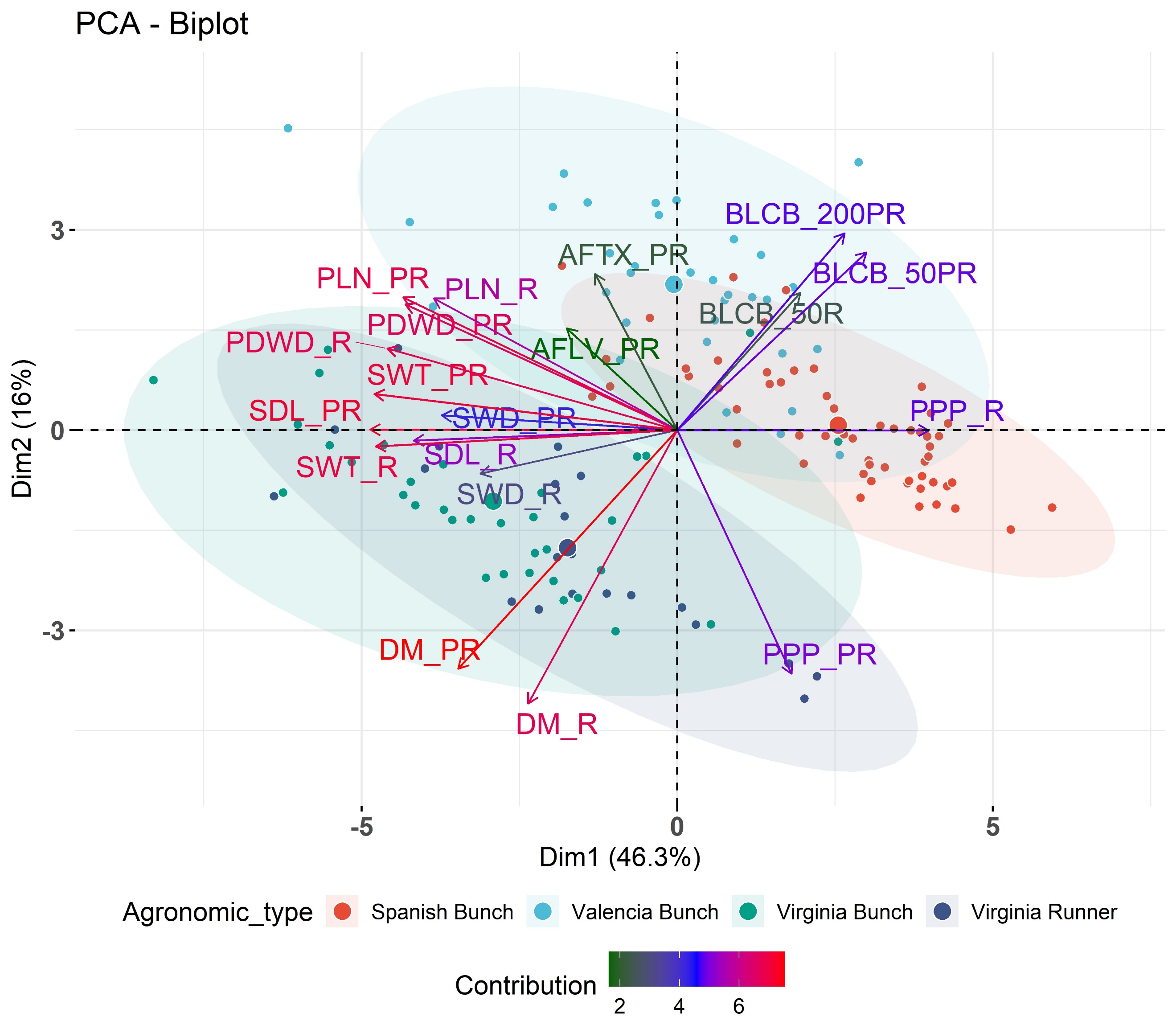
**Supplementary Figure S1. Principal component analysis for blanchability, aflatoxin and other agronomic traits.** Projection of the minicore collection on the first plane of principal component analysis using phenotypic data for blanchability, aflatoxin and other agronomic traits [DM (day to maturity), SWD (seed width), PPP (pods per plant), PDWD (pod width), SDL (seed length), SWT (seed weight), BLCB (Blanchability) (50 gm) (200 gm), PLN (plant length) (PR:post-rainy and R:rainy)] across different season and sample size. The first two components, PC1 and PC2, explain 62.3% of the variance between genotypes. Among the traits, BLCB and DM have been observed to be negatively associated.


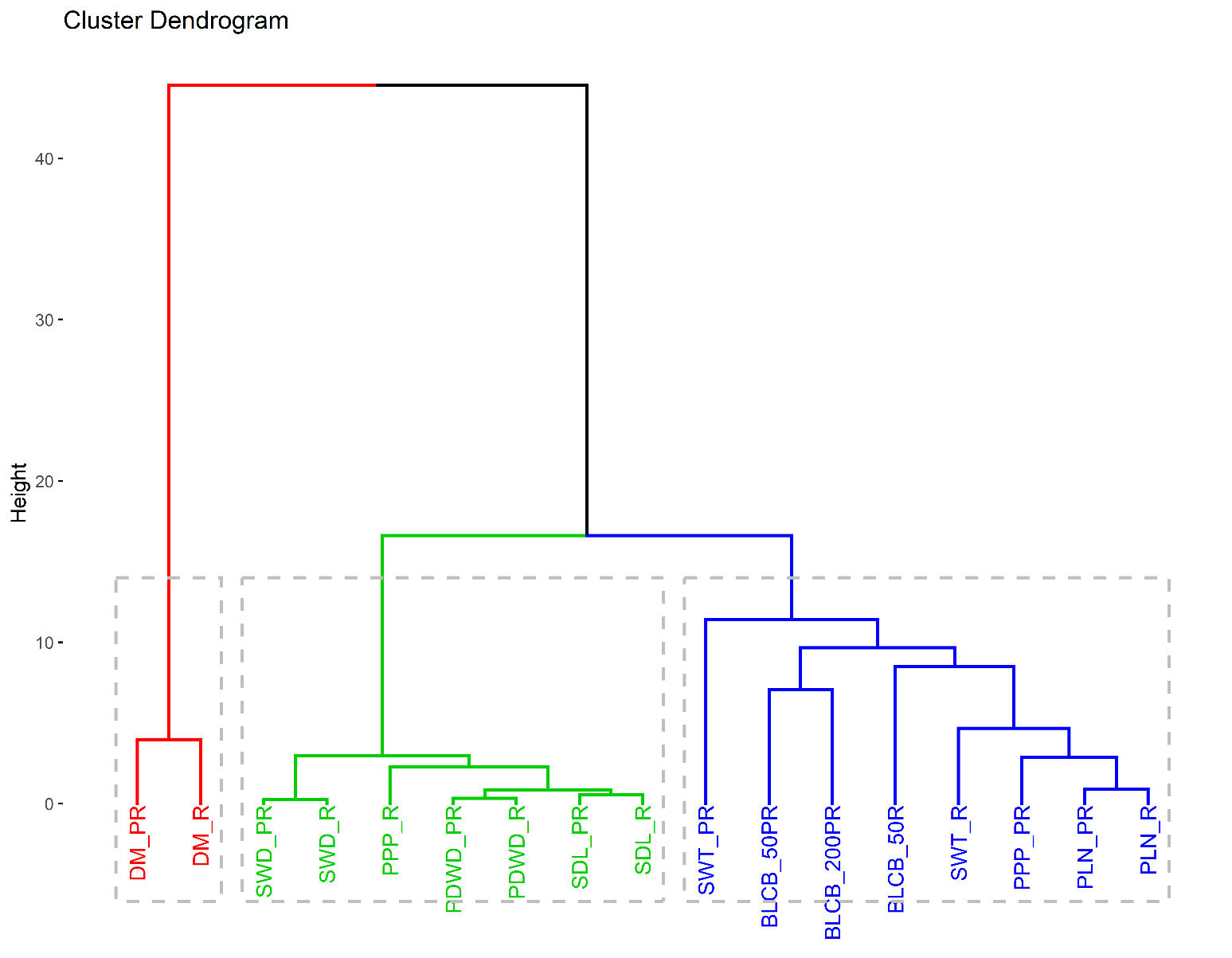
**Supplementary Figure S2: Hierarchical cluster analysis for blanchability and other agronomic traits** [DM (day to maturity), SWD (seed width), PPP (pods per plant), PDWD (pod width), SDL (seed length), SWT (seed weight), BLCB (Blanchability) (50 gm) (200 gm), PLN (plant length) (PR:post-rainy and R:rainy)] were grouped into three distinct clusters, are represented in different colors : **(1)** DM (PR and R); **(2)** SWD (PR and R),PPP (R), PDWD (PR and R), SDL (PR and R); **(3)** SWT (PR and R) (BLCB 50 (PR and R), 200 (PR), PPP (PR), PLN (PR and R)

**
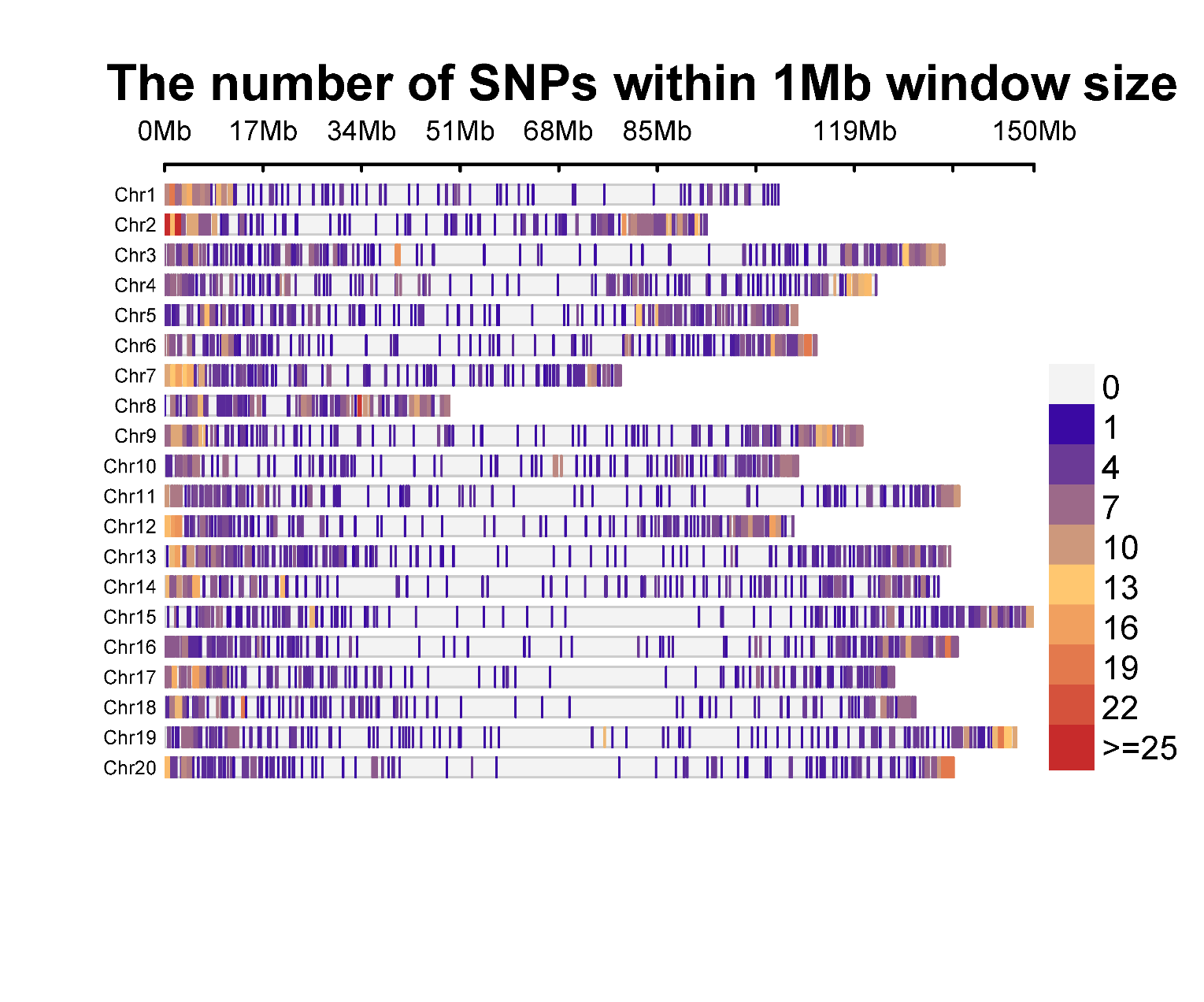
**

**Supplementary Figure S3: SNP density plot with 1Mb window size for 58 K ‘Axiom_*Arachis*’ array data.** Red-colored regions, indicating the highest SNP density, appear sparsely, particularly on Chr A02. There are also white gaps on chromosomes, signifying regions with low or no detected SNPs.


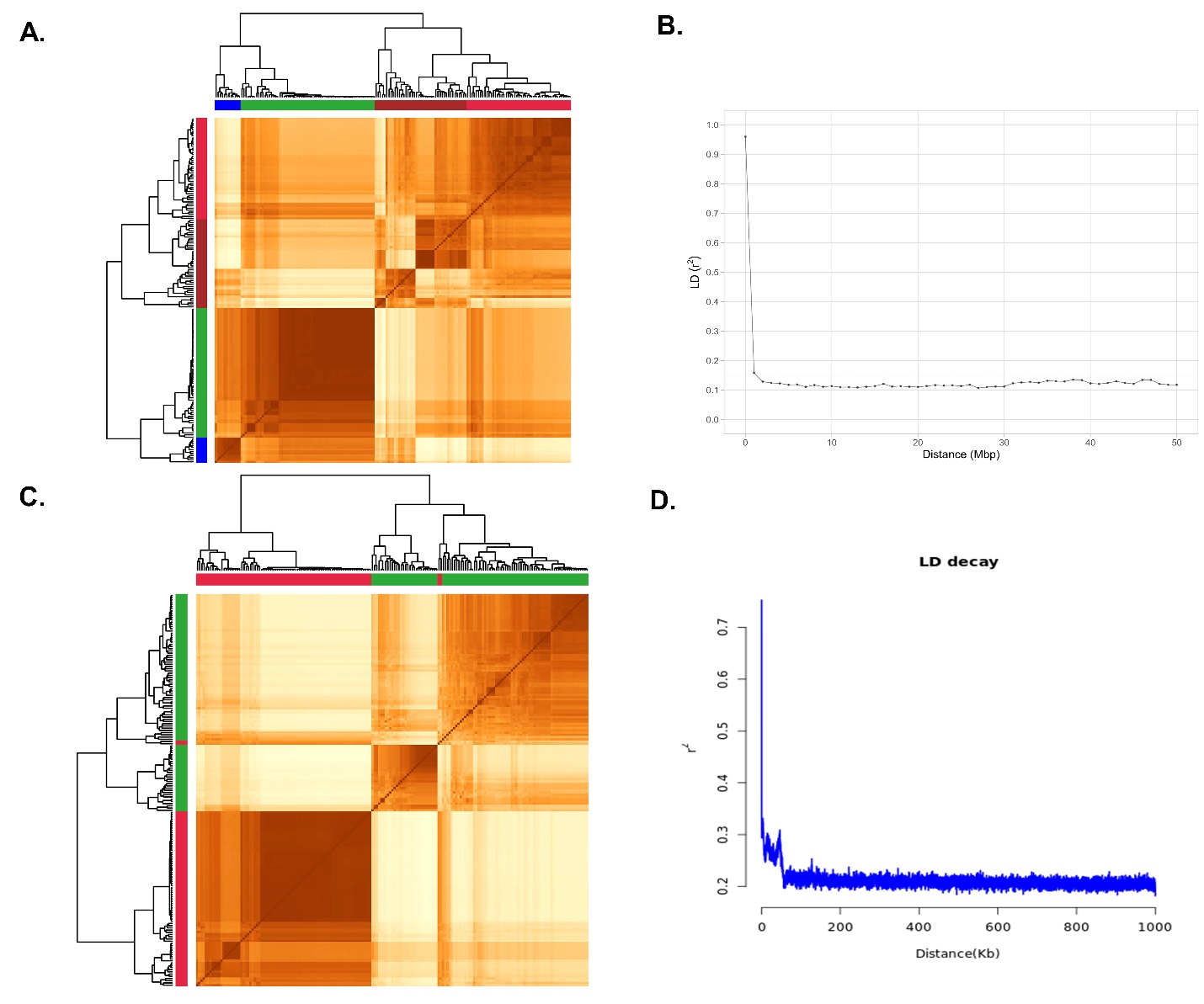


**Supplementary Figure S4: Population Structure analysis and genome-wide linkage disequilibrium, A.** Heat map of population structure, **B.** LD decay for groundnut minicore collection identified.

| 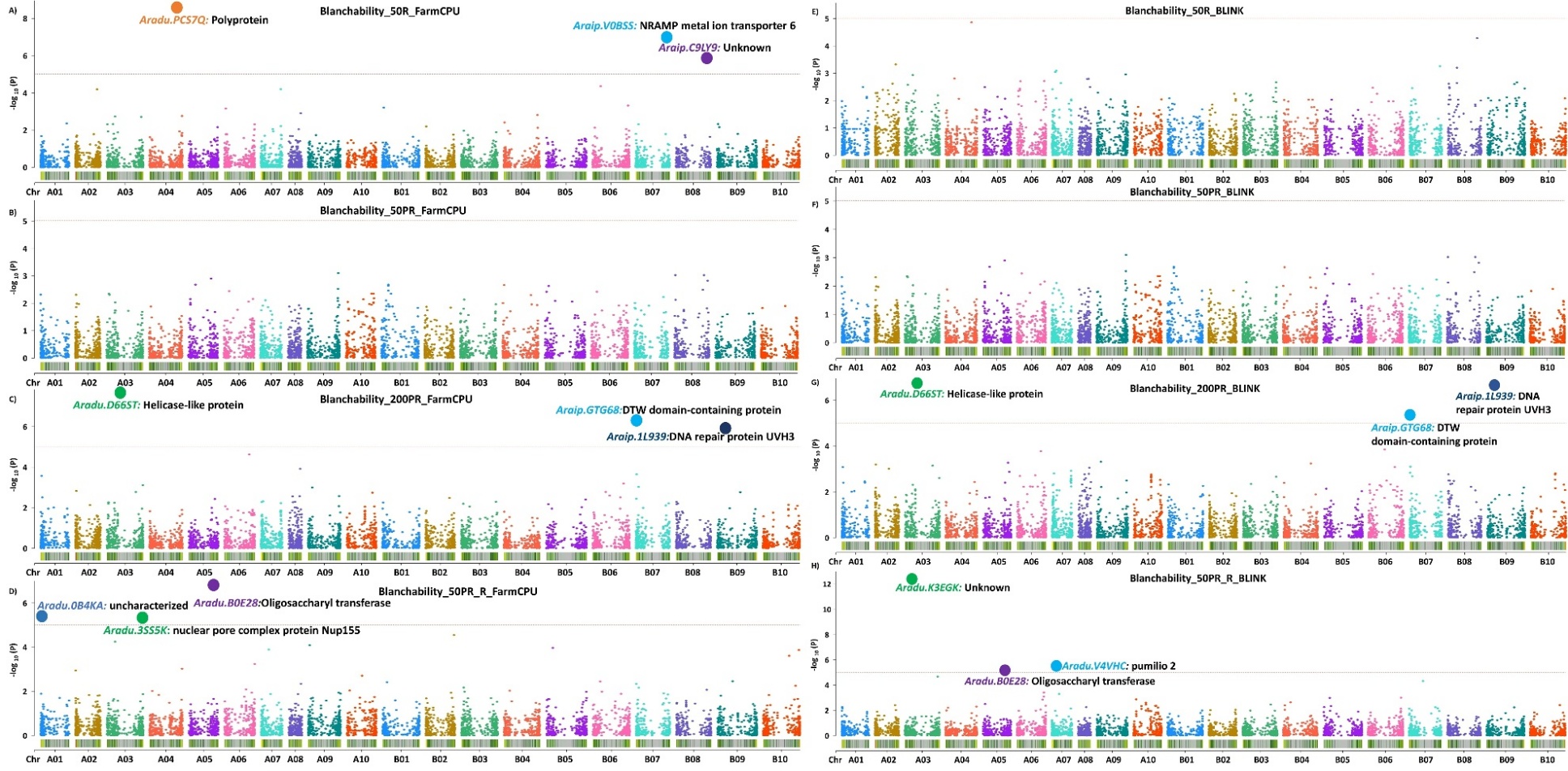 |
| --- |
| **Supplementary Figure S5:** **Manhattan plot representing the identified STAs associated with the blanchability on the basis A, B, C, D**: FarmCPU and **E, F, G, H**: BLINK model in GAPIT using 58K ‘Axiom_*Arachis*’ array data for Blanchability 50R, 50PR, 200PR and 50PR_R, respectively. The dots represent the significant STAs and the candidate genes. X-axis represents the chromosome number while the Y-axis represents -log_10_ (p) values of the SNPs detected. |
| 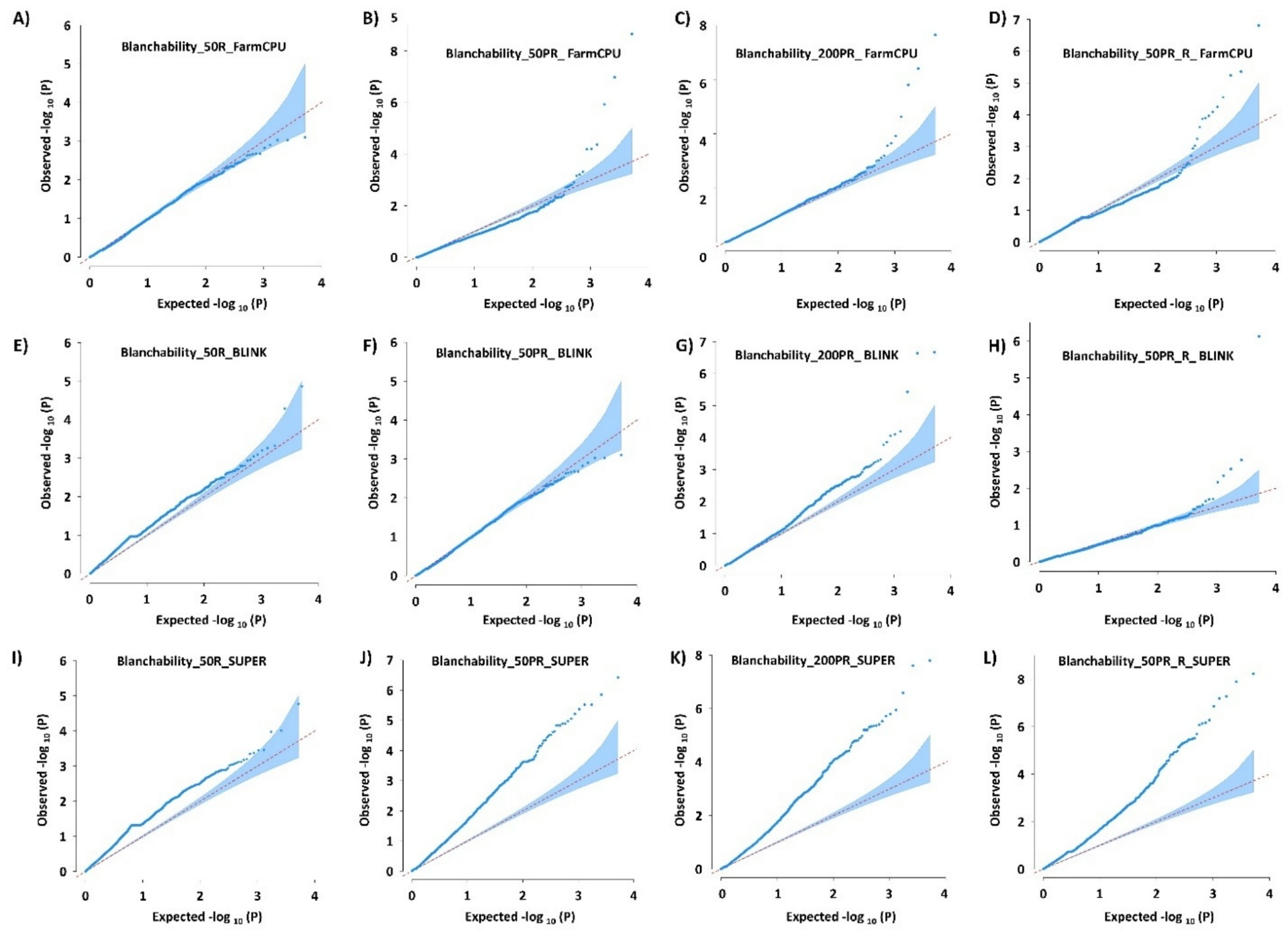 |
| **Supplementary. Figure S6:** **Quantile-Quantile (Q-Q) plot representing the identified STAs associated with the blanchability on the basis A, B, C, D**: Q-Q plot representing the identified STAs associated with the blanchability on the basis FarmCPU and **E, F, G, H**: BLINK, and I, J, K, L: SUPER model in GAPIT, using 58K ‘Axiom_*Arachis*’ array data for Blanchability 50R, 50PR, 200PR and 50PR_R, respectively. X-axis represents the expected -log_10_ (p) while the Y-axis represents the observed -log_10_ (p) values of the SNPs detected. |
| 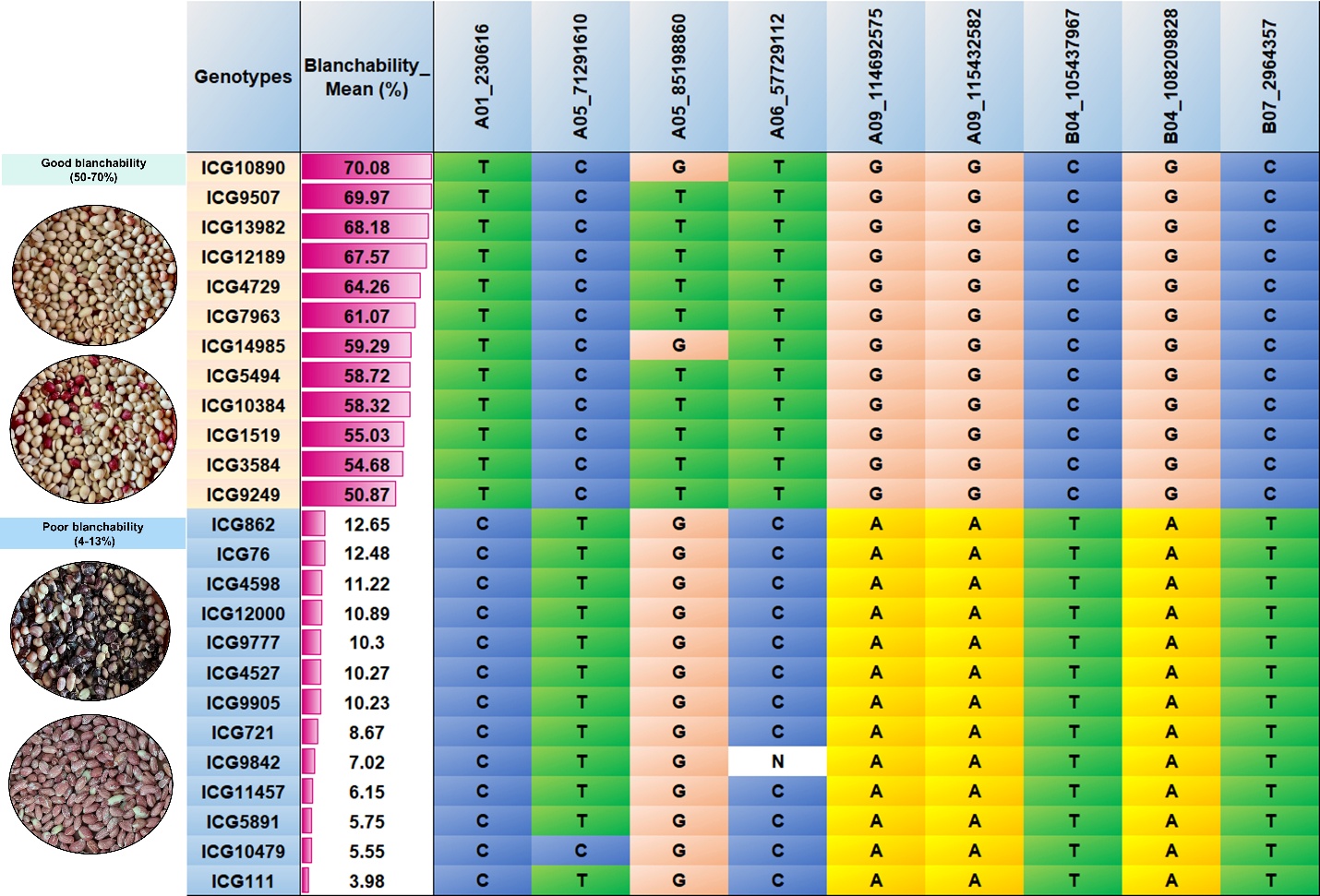 |
| **Supplementary Figure S7: Nine stable signiﬁcant polymorphic SNPs for blanchability in minicore collection,** including; A01_230616, A05_71291610, A05_85198860, A06_57729112, A09_114692575, A09_115432582, B04_105437967, B04_108209828, B07_2964357 |

**
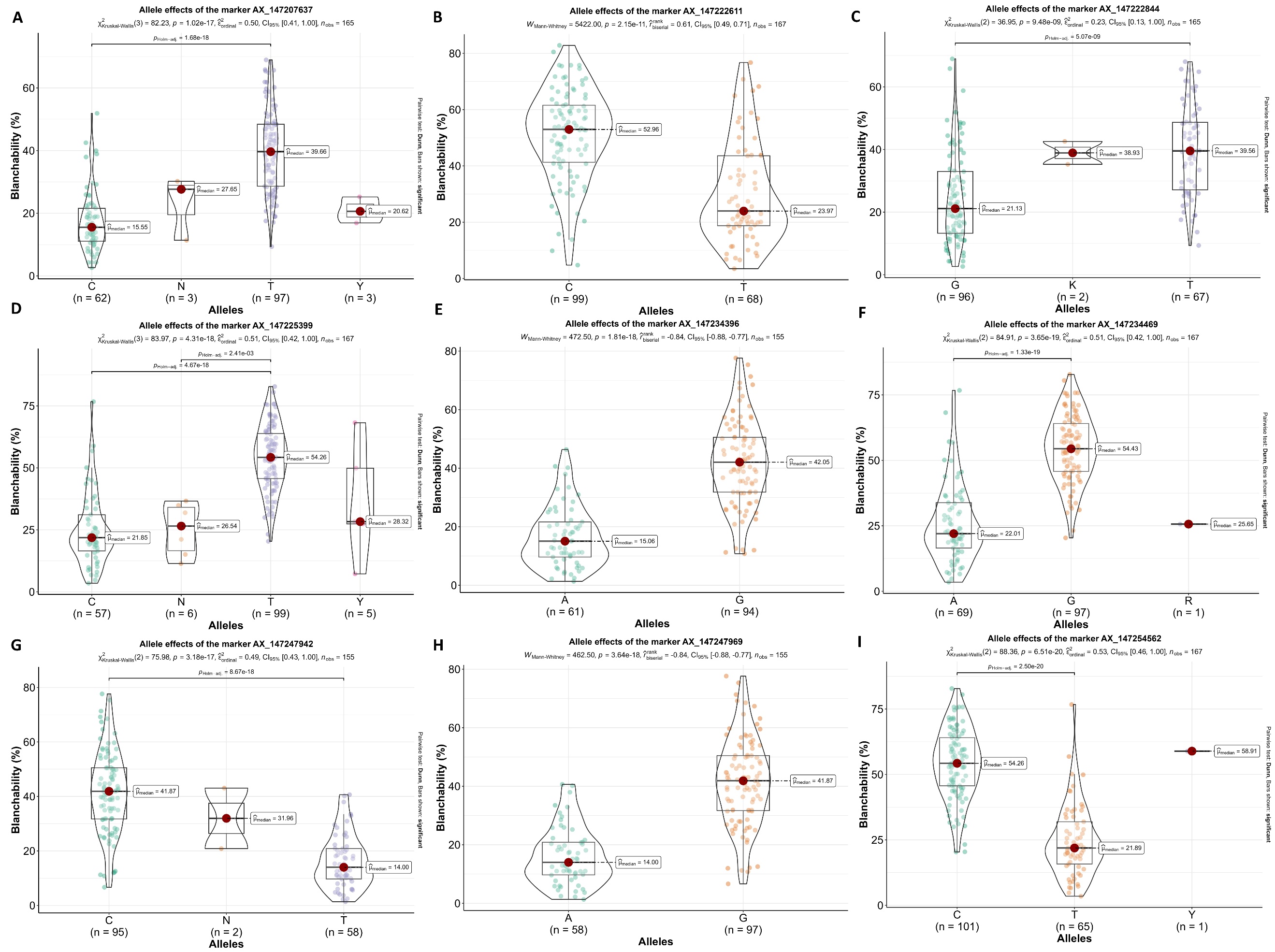
**

**Supplementary Figure S8: Allele-effect analysis for nine stable signiﬁcant SNPs** including **A.** A01_230616 (AX147207637), **B.** A05_71291610 (AX147222611), **C.** A05_85198860 (AX147222844), **D.** A06_57729112 (AX147225399), **E.** A09_114692575 (AX147234396), **F.** A09_115432582 (AX147234469), **G.** B04_105437967 (AX147247942), **H.** B04_108209828 (AX147247969), **I.** B07_2964357(AX147254562). The plot depicts the number of the alleles for each of the nine signiﬁcant SNPs in minicore collection, and the contribution of these alleles to the phenotypic variation observed blanchability**.**

.
